# Risk factors for early postoperative complications after minimally invasive adrenalectomy: a single-centre retrospective cohort study of more than 800 patients

**DOI:** 10.1007/s00464-026-13308-0

**Published:** 2026-08-31

**Authors:** Francesco Pennestrì, Antonio Laurino, Priscilla Francesca Procopio, Pierpaolo Gallucci, Annamaria Martullo, Francesca Prioli, Ilda Hoxhaj, Carmela De Crea, Marco Raffaelli

**Affiliations:** 1 UOC Chirurgia Endocrina e Metabolica, Fondazione Policlinico Universitario Agostino Gemelli IRCCS, L.go A. Gemelli 8, 00168 Rome, Italy; 2 Centro di Ricerca in Chirurgia delle Ghiandole Endocrine e dell’Obesità, Università Cattolica del Sacro Cuore, Rome, Italy

**Keywords:** Adrenalectomy, Minimally invasive surgery, Postoperative complications, Hypercortisolism, Conversion, Surgical risk

## Abstract

**Background:**

Minimally invasive adrenalectomy is associated with low postoperative morbidity, but early complications still occur and may be influenced by patient-related, endocrine, and operative factors. This study aimed to identify the factors associated with 30-day postoperative complications after minimally invasive adrenalectomy.

**Methods:**

This retrospective cohort study included adults undergoing minimally invasive adrenalectomy at a high-volume endocrine surgery referral centre between January 2012 and March 2026. The primary outcome was the occurrence of postoperative complications within 30 days after surgery. Univariable and multivariable logistic regression analyses were performed, with results expressed as odds ratios (ORs) with 95% confidence intervals (CIs). Model discrimination was assessed using the area under the receiver operating characteristic curve (AUC), internal validation used bootstrap resampling, and model fit was evaluated using the Hosmer–Lemeshow test.

**Results:**

Among 860 patients who underwent adrenal surgery, 798 (92.8%) were included. Overall, 70 (8.8%) patients developed 30-day postoperative complications. In the multivariable model, hypercortisolism was independently associated with postoperative complications compared with non-functioning lesions (OR 2.413, 95% CI 1.319–4.415; *p* = 0.004). Compared with ASA score 1, ASA score 3 was independently associated with postoperative complications (OR 1.972, 95% CI 1.074–3.620; *p* = 0.028), although the overall association for ASA score was borderline. Operative time (OR 1.008, 95% CI 1.003–1.014; *p* = 0.003), conversion to open surgery (OR 3.171, 95% CI 1.121–8.974; *p* = 0.030), and multiorgan resection (OR 3.698, 95% CI 1.178–11.609; *p* = 0.025) were also independently associated with postoperative complications. Surgical approach and laterality were not independent determinants of postoperative morbidity. The final explanatory model showed apparent discrimination, with an AUC of 0.754, decreasing to 0.730 after bootstrap optimism correction.

**Conclusions:**

These findings support a multidimensional interpretation of postoperative morbidity based on patient status, hormonal secretion pattern, and procedural complexity rather than on the minimally invasive approach alone. The model should be interpreted as explanatory and associational rather than as a dedicated preoperative prediction tool.

**Graphical abstract:**

**Figure:**
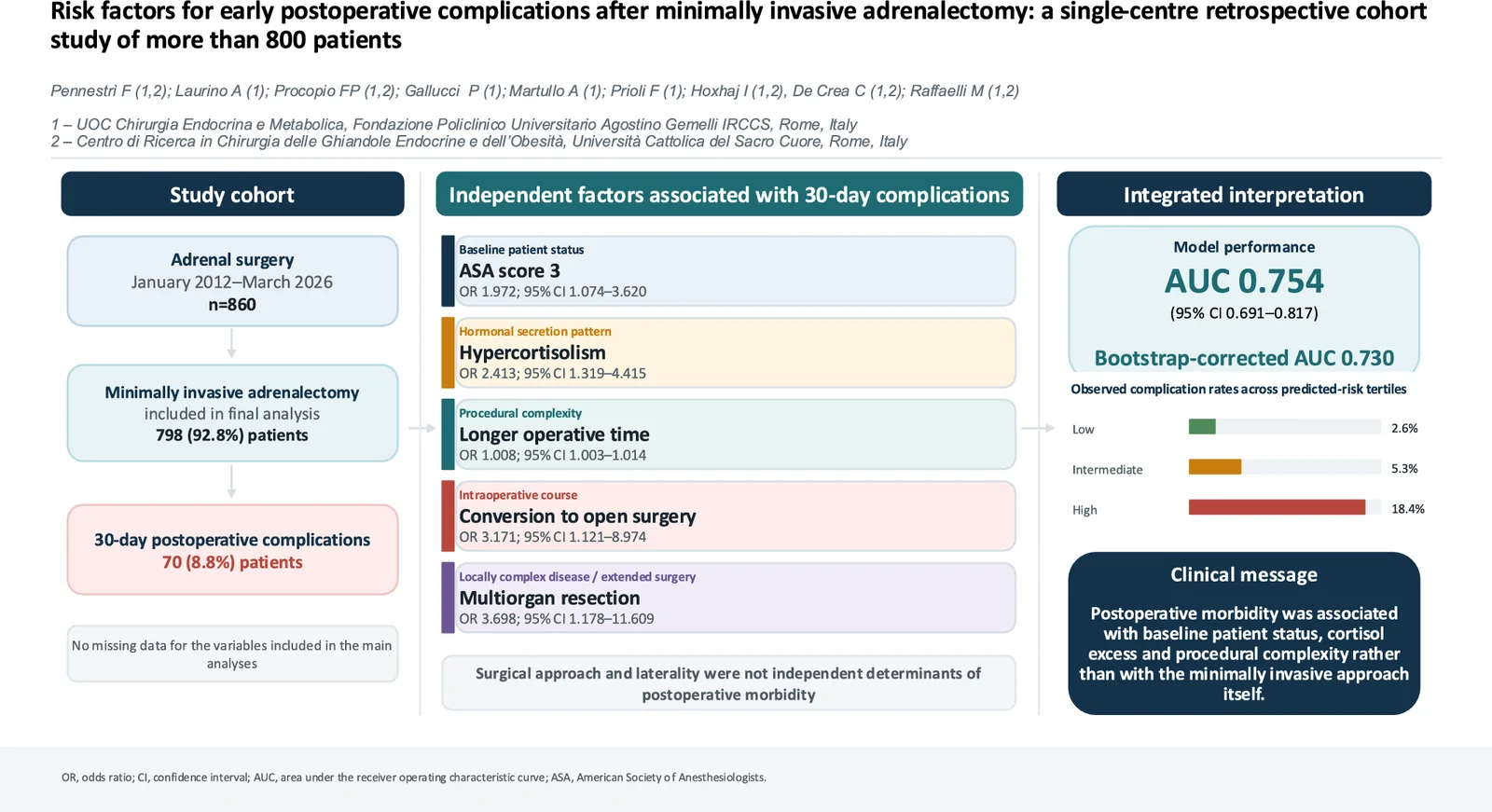

**Supplementary Information:**

The online version contains supplementary material available at https://doi.org/10.1007/s00464-026-13308-0.

The year 1992 marked a pivotal turning point in adrenal surgery, when Gagner et al. [1, 2] reported the first laparoscopic adrenalectomy performed through a lateral transabdominal approach. In the same year, Gaur [3] introduced balloon-assisted retroperitoneoscopy, providing the technical basis for endoscopic access to the retroperitoneal space. After early clinical application to adrenal surgery [4, 5], the posterior retroperitoneal approach was standardised and popularised by Walz et al. [6], establishing posterior retroperitoneoscopic adrenalectomy as a reproducible minimally invasive technique. Since then, several minimally invasive approaches for adrenalectomy have been developed [7]. Among these, laparoscopic lateral transabdominal adrenalectomy and posterior retroperitoneoscopic adrenalectomy have become the most widely adopted worldwide [7–9].

Robotic adrenalectomy subsequently emerged at the end of the 1990s, first with early robotic platforms and later with the Da Vinci Surgical System, with accumulating evidence supporting its feasibility and safety in selected patients [10–12]. However, most published experience with robotic adrenalectomy has involved the lateral transabdominal approach [13–16], whereas evidence on robotic posterior retroperitoneoscopic adrenalectomy remains limited, despite early feasibility reports and a small comparative experience with laparoscopic posterior retroperitoneoscopic adrenalectomy [17].

Compared to laparotomic surgery, minimally invasive adrenalectomy is associated with reduced postoperative pain, shorter hospital stay, faster recovery, lower wound-related morbidity, and improved cosmetic outcomes [18–22]. Consequently, it is now considered the standard of care for most benign adrenal tumours. In selected patients with adrenal-confined malignant lesions, this approach may also be considered when oncological principles can be respected, although tumour biology, local invasion, and avoidance of capsular disruption remain critical [23].

Despite the established perioperative benefits of minimally invasive adrenalectomy, postoperative complications remain a clinically relevant concern. Large institutional series and registry-based studies have reported postoperative complication rates ranging from approximately 5–13%: 8.4% in a 640-patient single-centre experience of laparoscopic adrenalectomy, 6.6% among 659 adrenalectomies recorded in the Scandinavian national registry, and 13.4% in a French single-centre academic series of 547 lateral transperitoneal laparoscopic adrenalectomies [24–26]. These data are difficult to compare because of heterogeneity in complication definitions, reporting thresholds, surgical indications, hormonal secretion patterns, operative approach, institutional volume, and surgeon experience [24, 27]. This is particularly relevant because adrenalectomy remains a relatively low-volume operation, even for endocrine surgeons, and outcomes are influenced by surgeon volume and centre expertise [28, 29]. Consequently, even large institutional series may include heterogeneous case mixes and few events within specific technical or disease-related subgroups.

Accordingly, early morbidity after minimally invasive adrenalectomy should be interpreted within a multifactorial framework, rather than as a function of surgical approach alone. Baseline patient status, hormonal secretion pattern, lesion-related features, and markers of procedural complexity may all contribute to postoperative complication risk. Clarifying their relative contribution may improve perioperative risk assessment and support the risk-adjusted interpretation of outcomes across different minimally invasive adrenalectomy approaches.

This single-centre retrospective study aimed to identify patient-, endocrine-, and procedure-related factors independently associated with 30-day postoperative complications after minimally invasive adrenalectomy in a tertiary endocrine surgery centre with long-standing experience in adrenal surgery. The analysis was intended to characterise these associations rather than to develop a dedicated preoperative prediction model.

This study has been reported in accordance with the STROCSS 2025 guidelines [30].

## Materials and methods

### Study design

We conducted a single-centre retrospective observational cohort study at a tertiary academic national and international referral centre for endocrine surgery in Rome, Italy. All consecutive adult patients scheduled for minimally invasive adrenalectomy between January 2012, corresponding to the introduction of robotic adrenal surgery into our clinical practice, and March 2026 were assessed for eligibility. 

Over the study period, the institutional adrenal surgery programme underwent progressive changes in case volume, surgical approach distribution, robotic platforms, and perioperative pathways. These temporal changes were considered in the interpretation of the findings and were further explored in sensitivity analyses.

Data were collected and managed using Microsoft Excel (Microsoft Corporation, Redmond, WA, USA) through a specifically designed de-identified database. All data were anonymised prior to analysis and handled in accordance with applicable data protection regulations.

Thirty-day postoperative follow-up data were obtained through scheduled outpatient assessments, review of inpatient and outpatient clinical records, and direct telephone contact when adequate follow-up information was not otherwise available. Patients with fewer than 30 days of documented postoperative follow-up were excluded. Data collection for the present study was completed on 30 April 2026.

The study was conducted in accordance with the principles of the Declaration of Helsinki and was approved by the Comitato Etico Territoriale Lazio Area 3–Roma (approval ID: 4853).

Inclusion criteria comprised adult patients planned for minimally invasive adrenalectomy. Exclusion criteria included age < 18 years, a preoperative indication for open adrenalectomy, and follow-up shorter than 30 days.

All patients provided written informed consent for the surgical procedure and, at the time of surgery, for the use of their anonymised clinical data for research and publication purposes.

### Data collection

Baseline characteristics comprised sex (as assigned at birth), age, and body mass index (BMI). Preoperative variables comprised hormonal secretion pattern, tumour side, and tumour size. Hypercortisolism was classified as mild autonomous cortisol secretion (MACS) or overt Cushing syndrome according to contemporary endocrine guidelines. MACS was defined by the absence of clinical features of overt Cushing syndrome together with a serum cortisol concentration >50 nmol/L (> 1.8 µg/dL) after the 1-mg overnight dexamethasone suppression test. Overt Cushing syndrome was diagnosed in patients with clinically apparent cortisol excess supported by biochemical evidence of endogenous hypercortisolism [31, 32]. Operative variables recorded included surgical approach, type of robotic platform used, operative time, intraoperative complications, and conversion to open surgery. Postoperative variables comprised histopathological findings, postoperative hospital stay, early postoperative complications, readmission, and mortality.

Operative time was defined as the time from skin incision to definitive wound closure. Intraoperative complications were considered as any adverse event occurring during surgery that had the potential to cause patient harm and required unplanned intraoperative actions. Postoperative complications were defined as any deviation from the expected postoperative course or any condition leading to prolonged hospitalisation within 30 days after surgery and were graded according to the Clavien–Dindo classification [33]. Thirty-day mortality included any death that occurred intraoperatively or within 30 days after surgery.

### Bias

Given the retrospective observational design, several measures were implemented to reduce potential sources of bias. Eligibility criteria were defined a priori and applied uniformly across the study period. Data were extracted from a dedicated institutional database using predefined variable definitions and were cross-checked against electronic clinical records, operative reports, and pathology reports to ensure completeness and consistency. Postoperative complications were reviewed and graded according to the Clavien–Dindo classification [33]. To reduce information bias, only patients with at least 30 days of postoperative follow-up were included. Potential confounding was addressed using multivariable logistic regression, with candidate variables selected according to predefined statistical criteria. Approach-specific analyses were considered exploratory due to the smaller subgroup size and fewer number of events. Residual confounding related to the retrospective design, temporal changes in practice, and non-random allocation of the surgical approach could not be fully excluded.

### Surgical technique

All procedures were performed by experienced endocrine surgeons; from 2017 onwards, operations were performed or supervised by the same expert endocrine surgeon (M.R.). The operating team consistently included an additional endocrine surgeon, a surgical resident, two dedicated scrub nurses, and an anaesthesiologist with specific expertise in adrenal surgery.

The learning curve for laparoscopic lateral transabdominal adrenalectomy and posterior retroperitoneoscopic adrenalectomy had been completed before the study period, indicating established surgical proficiency at the time of patient enrolment. The institutional adrenalectomy volume progressively increased over the study period, reaching approximately 100 procedures annually in recent years. The surgical approach was determined according to patient- and lesion-related factors, together with surgeon preference [34].

Starting in June 2016, in patients undergoing lateral transabdominal adrenalectomy, whenever technically feasible, a Pfannenstiel incision was preferentially used, particularly for larger lesions, as the specimen extraction site, to minimise the risk of extraction-site incisional hernia [35].

Robotic adrenalectomy was first performed in our centre in 2012 using the Da Vinci Si platform (Intuitive Surgical, Sunnyvale, CA, USA) and followed in 2016 by the Da Vinci Xi platform. HUGO™-RAS (Medtronic, Minneapolis, MN, USA), Versius® (CMR Surgical, Cambridge, UK), and Toumai^TM^ (MicroPort MedBot Group, Shanghai, China) platforms were introduced in 2022, 2024, and 2025, respectively. Until 2025, all robotic procedures were conducted via the lateral transabdominal approach. From 2025 onwards, a posterior retroperitoneoscopic approach was also adopted, initially using the Toumai^TM^ platform and subsequently the Da Vinci Xi system.

The surgical techniques for laparoscopic lateral transabdominal adrenalectomy, posterior retroperitoneoscopic adrenalectomy, and robotic lateral transabdominal adrenalectomy have been described previously [7].

The technique of robotic posterior retroperitoneoscopic adrenalectomy is as follows. The patient is placed in the prone position, supported by a Wilson frame. The operating table is flexed in a jack-knife configuration, with the back kept level to increase the space between the posterior costal margin and the iliac crest. Three robotic ports are used: one (8 mm or 10 mm) optical trocar and two 8 mm robotic working trocars, positioned medially and laterally to the camera port. For the camera port, trocar-in-trocar technique can be used, with the robotic camera trocar inserted through a 12 mm balloon trocar. The retroperitoneal space is accessed through a combination of blunt and sharp dissection of the abdominal wall, creating an initial working cavity that allows placement of the additional trocars under digital guidance, approximately 4–5 cm from the initial incision, along the mid-axillary line laterally and adjacent to the erector spinae muscle medially. Zuckerkandl’s fascia is identified and incised under direct endoscopic vision, after which the robotic platform is docked using three robotic arms. Subsequent surgical steps mirror those of the conventional posterior retroperitoneoscopic adrenalectomy. The retroperitoneal working space is maintained using carbon dioxide insufflation at a pressure of 10–20 mmHg.

The operative techniques were standardised within the institutional adrenal surgery programme, although refinements related to surgical approach, robotic platforms, and perioperative care occurred over the study period.

### Perioperative protocol

All patients underwent preoperative endocrine and anaesthetic assessment. Disease-specific medical preparation was performed when indicated, according to the underlying hormonal pattern. Perioperative antibiotic prophylaxis, venous thromboembolism prophylaxis, anaesthetic management, and postoperative clinical and biochemical monitoring followed standardised institutional protocols throughout the study period. Discharge criteria were based on institutional protocols and were progressively refined over time, as previously described [7, 34].

### Study endpoints

The primary outcome was the occurrence of any postoperative complication within 30 days after minimally invasive adrenalectomy, graded according to the Clavien–Dindo classification. The primary objective was to identify the factors independently associated with this outcome. The multivariable analysis was designed as an explanatory association model and was not intended to develop a preoperative clinical prediction score.

Secondary study endpoints included the exploratory assessment of these factors according to surgical procedure in the three subgroups with sufficient sample size and event distribution for regression analysis: laparoscopic lateral transabdominal adrenalectomy, laparoscopic posterior retroperitoneoscopic adrenalectomy, and robotic lateral transabdominal adrenalectomy. Given the smaller subgroup size and the expected low number of events, these analyses were considered supportive and were not intended to infer approach-specific superiority or inferiority.

In addition, a predefined subgroup analysis restricted to unilateral adrenalectomies was performed to evaluate the association between the side of surgery and early postoperative complications. Finally, cumulative sum (CUSUM) analysis was conducted to assess the temporal distribution of postoperative complications across the study period.

### Sample size

Because no reliable preliminary estimate of the 30-day postoperative complication rate was available for our institutional cohort, the sample-size calculation was based on the range most commonly reported in the literature. Assuming an expected complication rate of 5%–10%, a 2% absolute margin of error, and a 95% confidence level, the estimated required sample size ranged from 457 patients for an expected complication rate of 5% to 865 patients for an expected complication rate of 10%. The final study population therefore fell within this estimated range.

### Statistical analyses

Descriptive statistics were used to summarise patient characteristics. Continuous variables were expressed as median and interquartile range (IQR; Q1–Q3), whereas categorical variables were reported as frequencies and percentages. There were no missing data for the variables included in the analyses; therefore, no data imputation was required. The normality of continuous variables was assessed using the Shapiro–Wilk test. Categorical variables were compared using the chi-square test or Fisher’s exact test, as appropriate. Continuous variables were compared using Student’s t-test for normally distributed data or the Mann–Whitney U test for non-normally distributed variables. Univariable logistic regression was initially performed to assess the association between candidate variables and 30-day postoperative complications. Each covariate was initially assessed using univariable logistic regression. Variables with a *p* value < 0.20 at univariable analysis were entered into the multivariable logistic regression model, and backward stepwise selection was applied to identify the independently associated factors. The number of events per estimated regression coefficient was calculated by dividing the number of outcome events by the number of coefficients included in the final model, with categorical variables contributing one coefficient for each non-reference category. Linearity in the logit for continuous predictors retained in the final multivariable model was assessed using the Box–Tidwell approach. Model discrimination was evaluated using receiver operating characteristic curve analysis and expressed as the area under the receiver operating characteristic curve (AUC), with its 95% confidence interval. Internal validation was performed using 1000 bootstrap resamples. Optimism was estimated as the mean difference between model performance in each bootstrap sample and the performance of the corresponding bootstrap-fitted model when applied to the original dataset. The optimism-corrected AUC was obtained by subtracting the estimated optimism from the apparent AUC. Model calibration was assessed using the Hosmer–Lemeshow goodness-of-fit test. Univariable and multivariable logistic regression analyses estimated odds ratios (ORs) with corresponding 95% confidence intervals (CIs) for the association between covariates and early postoperative complications. To explore the cumulative risk profile generated by the final multivariable model, individual predicted probabilities of 30-day postoperative complications were derived from the logistic regression model and stratified into tertiles. Observed complication rates were compared across predicted-risk tertiles using the chi-square test, and the linear-by-linear association test was used to assess trend across ordered risk strata.

CUSUM analysis was performed to evaluate the temporal trend of postoperative complications across the study period. Case order was used as the time variable. Recursive cumulative sum testing for parameter stability and single-break structural change analysis using the Supremum Wald test were used to identify potential temporal inflection points and changes in the complication pattern throughout the study period. To assess the potential influence of temporal heterogeneity on the primary findings, two sensitivity analyses were performed by adding a temporal covariate to the final multivariable logistic regression model using the enter method. In the first analysis, surgical era was dichotomised according to the inflection identified by CUSUM analysis as procedures performed up to and including 2018 versus procedures performed from 2019 onwards. In the second analysis, chronological case order, ranging from 1 to 798, was entered as a continuous covariate. All other variables and reference categories were maintained as in the final primary model.

CUSUM analysis was conducted using Stata version 19 (StataCorp LP, College Station, TX, USA), while all other statistical analyses were performed using IBM SPSS Statistics version 31 (IBM Corp., Armonk, NY, USA) and R 4.3.1. Statistical analyses were performed by the study investigators.

All analyses were two-tailed, and statistical significance was set at *p *< 0.05.

## Results

### General characteristics

**Fig. 1:**
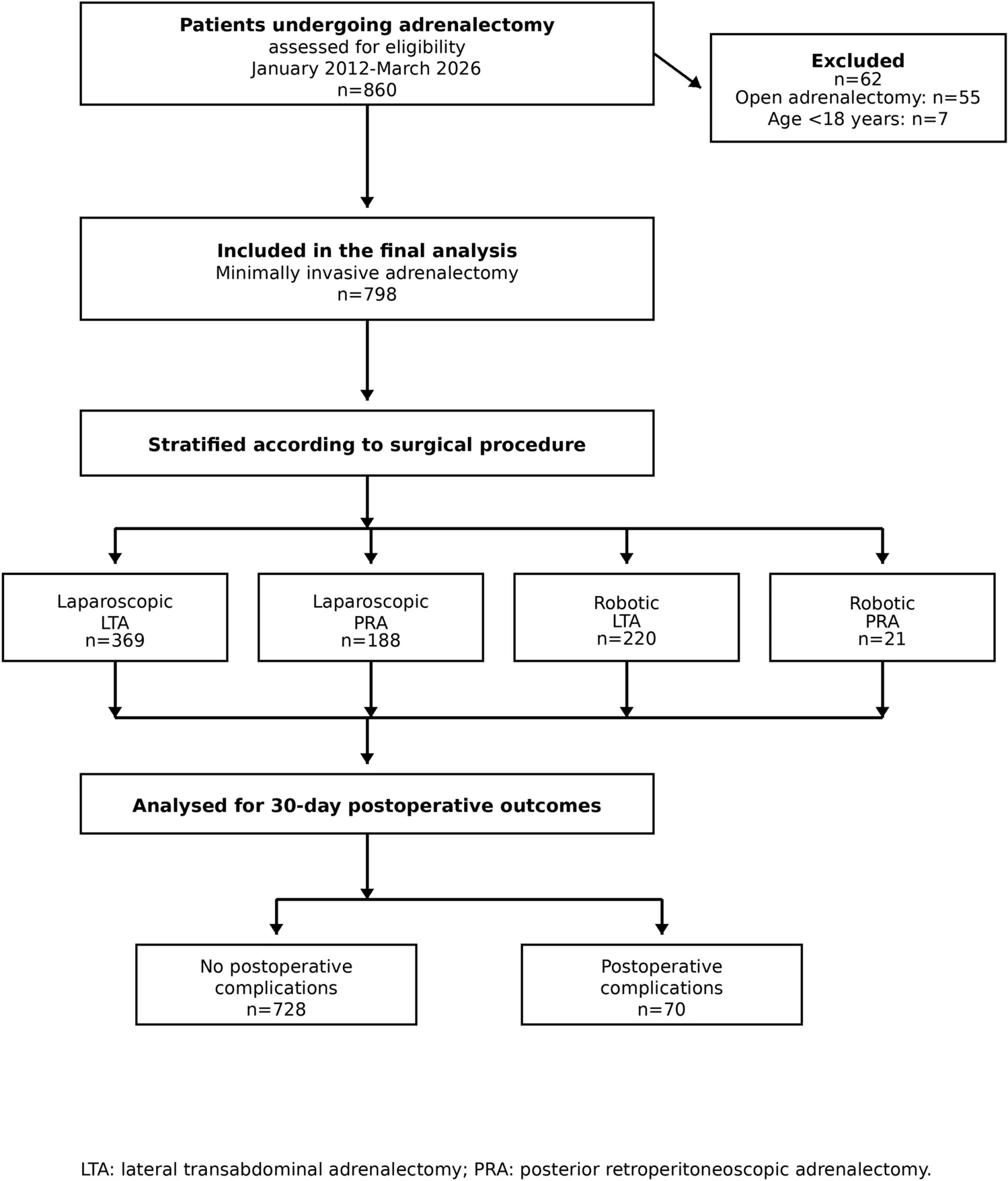
Flow diagram of patient selection and study population. Of 860 patients assessed for eligibility between January 2012 and March 2026, 62 were excluded (55 scheduled for primary open adrenalectomy and 7 aged < 18 years), leaving 798 patients who underwent minimally invasive adrenalectomy and were included in the final analysis. *LTA* lateral transabdominal adrenalectomy, *PRA* posterior retroperitoneoscopic adrenalectomy

Among 860 patients who underwent adrenal surgery between January 2012 and March 2026, 798 (92.8%) were included in the final analysis (Fig. 1). No missing data were recorded for the variables included in the main analyses. Preoperative, intraoperative, postoperative, and histopathological characteristics of the overall cohort are summarised in Table 1. Median age was 56.0 (47.0–66.0) years, and 514 (64.4%) patients were female. Median BMI was 25.4 (22.7–29.2) kg/m^2^. Hormonal hypersecretion was present in 454 (56.9%) patients, with hypercortisolism representing the most frequent pattern, occurring in 236 (29.6%) patients. Overall, 557 (69.8%) procedures were laparoscopic and 241 (30.2%) robotic. The surgical approach was lateral transabdominal in 589 (73.8%) procedures and posterior retroperitoneoscopic in 209 (26.2%). By surgical procedure, 369 (46.2%) patients underwent laparoscopic lateral transabdominal adrenalectomy, 188 (23.6%) underwent laparoscopic posterior retroperitoneoscopic adrenalectomy, 220 (27.6%) underwent robotic lateral transabdominal adrenalectomy, and 21 (2.6%) underwent robotic posterior retroperitoneoscopic adrenalectomy. Median operative time was 70.0 (50.0–105.0) minutes. Lymph node dissection was performed in 47 (5.9%) patients, and multiorgan resection in 19 (2.4%). Conversion to open surgery was required in 25 (3.1%) patients, and intraoperative complications occurred in 21 (2.6%). Thirteen conversions occurred during laparoscopic lateral transabdominal adrenalectomy, five during laparoscopic posterior retroperitoneoscopic adrenalectomy, and seven during robotic lateral transabdominal adrenalectomy; no conversions occurred during robotic posterior retroperitoneoscopic adrenalectomy. In laparoscopic lateral transabdominal adrenalectomy, conversion was required because of suspected or confirmed local infiltration (*n* = 8), inferior vena cava bleeding (*n* = 2), hepatomegaly (*n* = 1), haemodynamic instability (*n* = 1), or left renal artery bleeding (*n* = 1). In laparoscopic posterior retroperitoneoscopic adrenalectomy, the indications were technical difficulties (*n* = 2), haemodynamic instability (*n* = 1), intra-abdominal adhesions (*n* = 1), and renal parenchymal bleeding (*n* = 1). In robotic lateral transabdominal adrenalectomy, conversion was required because of suspected local infiltration (*n* = 4), intra-abdominal adhesions (*n* = 2), or inferior vena cava bleeding (*n* = 1).

**Table 1:** Preoperative, intraoperative, postoperative, and histopathological characteristics of the overall study cohort

Patients	798
Age (years)	56.0 (47.0–66.0)
Sex*	
Male	284 (35.6%)
Female	514 (64.4%)
BMI (kg/m^2^)	25.4 (22.7–29.2)
ASA score	
1	22 (2.8%)
2	610 (76.4%)
3	166 (20.8%)
Hormonal hypersecretion	
No	344 (43.1%)
Yes	454 (56.9%)
Pattern of hormonal hypersecretion	
Non-functioning	344 (43.1%)
Primary hyperaldosteronism	65 (8.1%)
Catecholamine hypersecretion	153 (19.2%)
Hypercortisolism	236 (29.6%)
Previous abdominal surgery	
No	475 (59.5%)
Yes	323 (40.5%)
Operative time (minutes)	70 (50–105)
Surgical technique	
Laparoscopic	557 (69.8%)
Robotic	241 (30.2%)
Surgical approach	
LTA	589 (73.8%)
PRA	209 (26.2%)
Surgical procedure	
Laparoscopic LTA	369 (46.2%)
Laparoscopic PRA	188 (23.6%)
Robotic LTA	220 (27.6%)
Robotic PRA	21 (2.6%)
Conversion	
No	773 (96.9%)
Yes	25 (3.1%)
Laterality	
Right	383 (48.0%)
Left	383 (48.0%)
Bilateral	32 (4.0%)
Extent of adrenal surgery	
Unilateral	766 (96.0%)
Bilateral	32 (4.0%)
Partial adrenalectomy	
No	786 (98.5%)
Yes	12 (1.5%)
Lymph node dissection	
No	751 (94.1%)
Yes	47 (5.9%)
Multiorgan resection	
No	779 (97.6%)
Yes	19 (2.4%)
Intraoperative blood transfusion	
No	794 (99.5%)
Yes	4 (0.5%)
Intraoperative complications	
No	777 (97.4%)
Yes	21 (2.6%)
ICU stay	
No	755 (94.6%)
Yes	43 (5.4%)
30-day postoperative complications	
No	728 (91.2%)
Yes	70 (8.8%)
Clavien–Dindo grade	
0	728 (91.2%)
I	14 (1.8%)
II	42 (5.3%)
IIIa	2 (0.2%)
IIIb	3 (0.4%)
IV	7 (0.8%)
V	2 (0.3%)
Mortality within 6 months from the index operation	4 (0.5%)
Postoperative hospital stay (days)	3.0 (2.0–4.0)
Lesion size (cm)	4.0 (3.0–6.0)
Pathological diagnosis	
Benign lesions	546 (68.4%)
Pheochromocytomas	169 (21.2%)
Adrenocortical neoplasms of uncertain malignant potential	15 (1.9%)
Adrenocortical carcinomas	25 (3.1%)
Adrenal metastases	37 (4.6%)
Other malignancies	6 (0.8%)

All continuous variables are reported as median and interquartile range (Q1–Q3), whereas categorical variables are reported as number and percentage

*LTA* lateral transabdominal adrenalectomy, *PRA* posterior retroperitoneoscopic adrenalectomy, *ICU* intensive care unit

^*^Sex as assigned at birth

Overall, 70 (8.8%) patients developed 30-day postoperative complications (Table 2). Median postoperative hospital stay was 3.0 (2.0–4.0) days. Five patients (0.6%) required hospital readmission within 30 days after surgery. The detailed Clavien–Dindo grade ≥II complications, stratified by pathological diagnosis and hormonal secretion pattern, are reported in Table 3.

**Table 2:** Comparison of preoperative, intraoperative, postoperative, and histopathological characteristics according to the occurrence of 30-day postoperative complications

	No complications	Complications	*p* value
Patients	728	70	–
Age (years)	56.0 (47.0–66.0)	58.0 (48.0–67.5)	0.252
Sex*			
Male	259 (35.6%)	25 (35.7%)	0.982
Female	469 (64.4%)	45 (64.3%)	
BMI (kg/m^2^)	25.4 (22.7–29.1)	26.0 (22.7–31.2)	0.460
ASA score			
1	19 (2.6%)	3 (4.3%)	**0.020**
2	566 (77.8%)	44 (62.9%)	
3	143 (19.6%)	23 (32.8%)	
Hormonal hypersecretion			
No	318 (43.7%)	26 (37.1%)	0.291
Yes	410 (56.3%)	44 (62.9%)	
Pattern of hormonal hypersecretion			
Non-functioning	318 (43.7%)	26 (37.1%)	**0.007**
Primary hyperaldosteronism	64 (8.8%)	1 (1.4%)	
Catecholamine hypersecretion	142 (19.5%)	11 (15.7%)	
Hypercortisolism	204 (28.0%)	32 (45.8%)	
Previous abdominal surgery			
No	440 (60.4%)	35 (50.0%)	0.089
Yes	288 (39.6%)	35 (50.0%)	
Operative time (minutes)	70.0 (49.0–100.0)	100.0 (70.0–153.5)	**< 0.001**
Surgical technique			
Laparoscopic	509 (69.9%)	48 (68.6%)	0.815
Robotic	219 (30.1%)	22 (31.4%)	
Surgical approach			
LTA	540 (74.2%)	49 (70.0%)	0.448
PRA	188 (25.8%)	21 (30.0%)	
Surgical procedure			
Laparoscopic LTA	340 (46.7%)	29 (41.4%)	0.839
Laparoscopic PRA	169 (23.2%)	19 (27.1%)	
Robotic LTA	200 (27.5%)	20 (28.6%)	
Robotic PRA	19 (2.6%)	2 (2.9%)	
Conversion			
No	712 (97.8%)	61 (87.1%)	**< 0.001**
Yes	16 (2.2%)	9 (12.9%)	
Laterality			
Right	359 (49.3%)	24 (34.3%)	**< 0.001**
Left	345 (47.4%)	38 (54.3%)	
Bilateral	24 (3.3%)	8 (11.4%)	
Extent of adrenal surgery			
Unilateral	704 (96.7%)	62 (88.6%)	**< 0.001**
Bilateral	24 (3.3%)	8 (11.4%)	
Partial adrenalectomy			
No	716 (98.4%)	70 (100%)	0.279
Yes	12 (1.6%)	0 (0%)	
Lymph node dissection			
No	689 (94.6%)	62 (88.6%)	**0.039**
Yes	39 (5.4%)	8 (11.4%)	
Multiorgan resection			
No	717 (98.5%)	62 (88.6%)	**< 0.001**
Yes	11 (1.5%)	8 (11.4%)	
Intraoperative blood transfusion			
No	727 (99.9%)	67 (95.7%)	**< 0.001**
Yes	1 (0.1%)	3 (4.3%)	
Intraoperative complications			
No	711 (97.7%)	66 (94.3%)	0.092
Yes	17 (2.3%)	4 (5.7%)	
ICU stay			
No	697 (95.7%)	58 (82.9%)	**< 0.001**
Yes	31 (4.3%)	12 (17.1%)	
Postoperative hospital stay (days)	3.0 (2.0–4.0)	7.0 (5.0–9.0)	**< 0.001**
Lesion size (cm)	4.0 (3.0–6.0)	4.2 (2.9–5.6)	0.919
Pathological diagnosis			
Benign lesions	502 (69.0%)	44 (62.9%)	0.371
Pheochromocytomas	155 (21.3%)	14 (20.0%)	
Adrenocortical neoplasms of uncertain malignant potential	13 (1.8%)	2 (2.9%)	
Adrenocortical carcinomas	20 (2.7%)	5 (7.1%)	
Adrenal metastases	33 (4.5%)	4 (5.7%)	
Other malignancies	5 (0.7%)	1 (1.4%)	

*LTA* lateral transabdominal adrenalectomy, *PRA* posterior retroperitoneoscopic adrenalectomy, *ICU* intensive care unit

All continuous variables are reported as median and interquartile range (Q1–Q3), whereas categorical variables are reported as number and percentage. Bold values indicate statistical significance at *p* < 0.05

*Sex as assigned at birth

**Table 3:** Detailed 30-day postoperative complications graded Clavien–Dindo ≥II, stratified by pathological diagnosis and hormonal secretion pattern

A. Pathological diagnosis							
Complication	Grade	Benign lesions	Pheochromocytomas	AUNMP	ACC	Adrenal metastases	Other malignancies
Atrial fibrillation	II	1	1	0	0	1	0
Bleeding	II	3	0	0	1	0	0
Fever	II	5	2	0	1	0	0
Intraparenchymal renal bleeding	II	1	0	0	0	0	0
Lymphatic fistula	II	3	0	0	0	0	0
Lymphatic fistula with superficial surgical-site infection	II	0	1	0	0	0	0
Non-ST-elevation myocardial infarction	II	0	1	0	0	0	0
Pancreatitis	II	0	0	0	0	0	1
Pleural effusion	II	2	0	0	0	0	0
Pneumonia	II	7	1	0	2	0	0
Polyuria	II	1	0	0	0	0	0
Renal ischaemia	II	0	0	0	0	1	0
Sepsis due to Staphylococcus aureus	II	0	1	0	0	0	0
Superficial surgical-site infection	II	2	1	0	0	0	0
Urinary tract infection	II	1	1	0	0	0	0
Intraparenchymal renal bleeding with lymphatic fistula	IIIa	1	0	0	0	0	0
Subcapsular hepatic haematoma with arterioportal fistula	IIIa	1	0	0	0	0	0
Perforated gastric ulcer	IIIb	0	0	1	0	0	0
Trocar-site bleeding	IIIb	2	0	0	0	0	0
Acute respiratory failure	IV	2	0	0	0	0	0
Bleeding	IV	1	1	0	0	1	0
Perforated gastric ulcer	IV	1	0	0	0	0	0
Splenic rupture	IV	0	0	1	0	0	0
Acute myocardial infarction	V	1	0	0	0	0	0
Haemoperitoneum	V	1	0	0	0	0	0

B. Hormonal secretion pattern					
Complication	Grade	Non-functioning	Primary hyperaldosteronism	Catecholamine hypersecretion	Hypercortisolism
Atrial fibrillation	II	1	0	1	1
Bleeding	II	2	0	0	2
Fever	II	3	0	0	5
Intraparenchymal renal bleeding	II	0	0	0	1
Lymphatic fistula	II	1	0	0	2
Lymphatic fistula with superficial surgical-site infection	II	0	0	1	0
Non-ST-elevation myocardial infarction	II	0	0	1	0
Pancreatitis	II	1	0	0	0
Pleural effusion	II	2	0	0	0
Pneumonia	II	3	1	0	6
Polyuria	II	1	0	0	0
Renal ischaemia	II	1	0	0	0
Sepsis due to Staphylococcus aureus	II	0	0	1	0
Superficial surgical-site infection	II	2	0	1	0
Urinary tract infection	II	1	0	1	0
Intraparenchymal renal bleeding with lymphatic fistula	IIIa	0	0	0	1
Subcapsular hepatic haematoma with arterioportal fistula	IIIa	0	0	0	1
Perforated gastric ulcer	IIIb	1	0	0	0
Trocar-site bleeding	IIIb	0	0	0	2
Acute respiratory failure	IV	1	0	0	1
Bleeding	IV	1	0	1	1
Perforated gastric ulcer	IV	0	0	0	1
Splenic rupture	IV	1	0	0	0
Acute myocardial infarction	V	0	0	0	1
Haemoperitoneum	V	0	0	0	1

*AUNMP* adrenocortical neoplasm of uncertain malignant potential, *ACC* adrenocortical carcinoma

Data are reported as number of patients. Each patient is classified according to the highest Clavien–Dindo grade recorded within 30 days after surgery

To further characterise complication patterns, postoperative events were classified as medical or surgical/procedure-related and analysed according to the presence of hypercortisolism (Table S5, Supplementary Materials). When all Clavien–Dindo grades were considered, patients with hypercortisolism had a higher incidence of postoperative complications than patients with other hormonal secretion patterns (32/236, 13.6% vs 38/562, 6.8%; OR 2.16, 95% CI 1.32–3.56; *p* = 0.004). Medical complications occurred in 14/236 (5.9%) and 22/562 (3.9%) patients, respectively (OR 1.55, 95% CI 0.78–3.08; *p* = 0.261), whereas surgical or procedure-related complications occurred in 18/236 (7.6%) and 16/562 (2.8%) patients, respectively (OR 2.82, 95% CI 1.41–5.63; *p* = 0.004). Similar findings were observed when the analysis was restricted to Clavien–Dindo grade ≥II events: clinically relevant complications occurred in 26/236 (11.0%) patients with hypercortisolism and 30/562 (5.3%) patients with other hormonal secretion patterns (OR 2.20, 95% CI 1.27–3.80; *p* = 0.006). Medical complications occurred in 13/236 (5.5%) versus 18/562 (3.2%) patients (OR 1.76, 95% CI 0.85–3.66; *p* = 0.158), whereas surgical or procedure-related complications occurred in 13/236 (5.5%) versus 12/562 (2.1%) patients (OR 2.67, 95% CI 1.20–5.95; *p* = 0.023).

Additional organ resection was performed in 19 patients (2.4%) and included partial nephrectomy (*n* = 10), nephrectomy (*n* = 1), limited liver resection (*n* = 6), pancreatic resection (*n* = 1), and distal pancreatectomy with splenectomy (*n* = 1). Thirteen procedures were planned preoperatively based on imaging, whereas six were undertaken according to intraoperative assessment. Detailed case-by-case characteristics of these procedures are reported in Table 4.

**Table 4:** Details of additional organ resections performed during minimally invasive adrenalectomy (*n* = 19)

#	Additional organ resection	R/L side	Indication	Planned	Pathological invasion
1	Limited liver resection	R	Partial intrahepatic location of the adrenal lesion	No	No
2	Nephrectomy	L	Pheochromocytoma with suspected renal and renal vascular involvement	Yes	Renal
3	Partial nephrectomy	R	Pheochromocytoma with suspected renal involvement	No	No
4	Partial nephrectomy	L	Suspected adrenal metastasis	No	No
5	Pancreatic resection	L	Suspected ACC with possible pancreatic involvement	Yes	No
6	Partial nephrectomy	L	Suspected adrenal metastasis with possible renal involvement	Yes	Microscopic renal invasion
7	Limited liver resection	R	Suspected adrenal metastasis with possible hepatic involvement	Yes	Microscopic liver invasion
8	Distal pancreatectomy + splenectomy	L	Malignant adrenal lesion with suspected local invasion	Yes	Pancreas and spleen
9	Limited liver resection	R	Pheochromocytoma with suspected hepatic involvement	Yes	No
10	Limited liver resection	L	Adrenal metastasis with a suspicious hepatic lesion	Yes	No
11	Partial nephrectomy	L	Pheochromocytoma with suspected renal involvement	Yes	No
12	Partial nephrectomy	L	Suspected adrenal malignancy with possible renal involvement	Yes	No
13	Limited liver resection	R	Pheochromocytoma with suspected hepatic involvement	No	No
14	Partial nephrectomy	L	Suspected ACC with possible renal involvement	Yes	Microscopic renal capsular invasion
15	Partial nephrectomy	R	Suspected adrenal malignancy with possible renal involvement	No	No
16	Limited liver resection	R	Suspected ACC with a suspicious hepatic lesion	Yes	No
17	Partial nephrectomy	R	Pheochromocytoma with suspected renal involvement	No	No
18	Partial nephrectomy	L	Pheochromocytoma with suspected renal involvement	Yes	Microscopic renal capsular invasion
19	Partial nephrectomy	L	Pheochromocytoma with suspected renal involvement	Yes	No

*ACC* adrenocortical carcinoma, *L* left, *R* right

Preoperative planning was defined by the anticipated need for additional organ resection on the basis of preoperative imaging and multidisciplinary assessment. Histopathological invasion was considered confirmed when microscopic infiltration of the additionally resected organ or structure was documented. None of the additional resections was performed to manage an intraoperative injury

On final histopathological examination, benign lesions were diagnosed in 546 (68.4%) patients, pheochromocytomas in 169 (21.2%), adrenocortical neoplasms of uncertain malignant potential in 15 (1.9%), adrenocortical carcinomas in 25 (3.1%), adrenal metastases in 37 (4.6%), and other malignancies in 6 (0.8%). Histopathological confirmation of invasion of the additionally resected organ or structure was observed in six patients.

The distribution of robotic adrenalectomies according to the robotic platform used and surgical approach is reported in Table S1 (Supplementary Materials). Characteristics stratified by surgical procedure and those restricted to unilateral adrenalectomy are provided in Tables S2 and S3 (Supplementary Materials), respectively. Detailed descriptions of intraoperative complications, additional details on 30-day postoperative complications, and mortality within 6 months are also provided in the Supplementary Materials.

### Primary outcome

The comparative analysis between patients with and without 30-day postoperative complications is shown in Table 2. Patients who developed complications differed significantly in ASA score and hormonal secretion pattern, and had higher rates of conversion to open surgery, bilateral adrenalectomy, lymph node dissection, multiorgan resection, intraoperative blood transfusion, and postoperative intensive care unit stay. They also had longer operative time and longer postoperative hospital stay. No significant differences were observed for sex, BMI, surgical technique, surgical approach, surgical procedure, lesion size, or pathological diagnosis. Table 5 shows the univariable and multivariable logistic regression analyses of factors associated with 30-day postoperative complications in the overall cohort. At univariable analysis, ASA score was associated with 30-day postoperative complications as an overall variable (*p* = 0.022), with ASA score 3 showing a borderline category-level association compared with ASA score 1 (OR 1.797, 95% CI 1.001–3.225; *p* = 0.050). Hormonal secretion pattern was also associated with the outcome (*p* = 0.015), with hypercortisolism significantly associated with 30-day postoperative complications compared with non-functioning lesions (OR 1.919, 95% CI 1.111–3.314; *p* = 0.019). Operative time (OR 1.012, 95% CI 1.008–1.016; *p* < 0.001), conversion to open surgery (OR 6.566, 95% CI 2.785–15.477; *p* < 0.001), bilateral adrenalectomy (OR 3.785, 95% CI 1.632–8.778; *p* = 0.002), lymph node dissection (OR 2.280, 95% CI 1.020–5.093; *p* = 0.045), multiorgan resection (OR 8.411, 95% CI 3.263–21.861; *p* < 0.001), intraoperative blood transfusion (OR 32.552, 95% CI 3.340–317.228; *p* = 0.003), and intensive care unit stay (OR 4.652, 95% CI 2.268–9.540; *p* < 0.001) were also associated with the outcome. Previous abdominal surgery and intraoperative complications showed borderline trends at univariable analysis but did not reach statistical significance.

**Table 5:** Univariable and multivariable logistic regression analyses of factors associated with 30-day postoperative complications in the overall study cohort

	Univariable analysis	Multivariable analysis
Age (years)	1.010 (0.991–1.028) *p* = 0.308	
Sex*		
Male (reference)		
Female	0.994 (0.596–1.659) *p* = 0.982	
BMI (kg/m^2^)	1.021 (0.978–1.066) *p* = 0.339	
ASA score	***p* = 0.022**	*p* = 0.066
1 (reference)		
2	1.013 (0.406–2.531) *p* = 0.978	0.650 (0.250–1.689) *p* = 0.377
3	**1.797 (1.001–3.225) *p* = 0.050**	**1.972** **(1.074–3.620) *p*** = **0.028**
Pattern of hormonal hypersecretion	***p* = 0.015**	***p* = 0.004**
Non-functioning (reference)		
Primary hyperaldosteronism	0.184 (0.025–1.434) *p* = 0.108	0.330 (0.043–2.529) *p* = 0.286
Catecholamine hypersecretion	0.947 (0.456–1.970) *p* = 0.885	0.868 (0.383–1.968) *p* = 0.734
Hypercortisolism	**1.919 (1.111–3.314) *p* = 0.019**	**2.413 (1.319–4.415) *p* = 0.004**
Previous abdominal surgery		
No (reference)		
Yes	**1.528 (0.935–2.498) *p* = 0.091**	1.441 (0.849–2.446) *p* = 0.176
Operative time (minutes)	**1.012 (1.008–1.016) *p* < 0.001**	**1.008 (1.003–1.014) *p* = 0.003**
Surgical procedure	*p* = 0.839	
Laparoscopic LTA (reference)		
Laparoscopic PRA	1.318 (0.718–2.419) *p* = 0.373	
Robotic LTA	1.172 (0.646–2.127) *p* = 0.601	
Robotic PRA	1.234 (0.274–5.562) *p* = 0.784	
Conversion		
No (reference)		
Yes	**6.566 (2.785–15.477) *p* < 0.001**	**3.171 (1.121–8.974) *p* = 0.030**
Extent of adrenal surgery		
Unilateral (reference)		
Bilateral	**3.785 (1.632–8.778) *p* = 0.002**	1.852 (0.656–5.229) *p* = 0.244
Partial adrenalectomy		
No (reference)		
Yes	Not estimable	
Lymph node dissection		
No (reference)		
Yes	**2.280 (1.020–5.093) *p* = 0.045**	0.948 (0.330–2.725) *p* = 0.921
Multiorgan resection		
No (reference)		
Yes	**8.411 (3.263–21.861) *p* < 0.001**	**3.698 (1.178–11.609) *p* = 0.025**
Intraoperative blood transfusion		
No (reference)		
Yes	**32.552 (3.340–317.228) *p* = 0.003**	4.673 (0.156–137.736) *p* = 0.375
Intraoperative complications		
No (reference)		
Yes	**2.535 (0.829–7.753) *p* = 0.103**	0.449 (0.098–2.048) *p* = 0.303
ICU stay		
No (reference)		
Yes	**4.652 (2.268–9.540) *p* < 0.001**	2.229 (0.963–5.159) *p* = 0.061
Lesion size (cm)	1.001 (0.993–1.010) *p* = 0.751	
Pathological diagnosis	*p* = 0.412	
Benign lesions (reference)		
Pheochromocytomas	1.030 (0.550–1.931) *p* = 0.925	
Adrenocortical neoplasms of uncertain malignant potential	1.755 (0.384–8.027) *p* = 0.468	
Adrenocortical carcinomas	2.852 (1.021–7.968) *p* = 0.046	
Adrenal metastases	1.383 (0.468–4.082) *p* = 0.557	
Other malignancies	2.282 (0.261–19.966) *p* = 0.456	

*LTA* lateral transabdominal adrenalectomy, *PRA* posterior retroperitoneoscopic adrenalectomy, *ICU* intensive care unit

The values reported in the table are odds ratios, with 95% confidence intervals and corresponding *p*-values. In the univariable analysis, bold values indicate variables meeting the predefined *p* < 0.20 criterion for inclusion in the multivariable model; in the multivariable analysis, bold values indicate statistical significance at *p* < 0.05

Variables not retained in the final multivariable model are left blank

^*^Sex as assigned at birth

In the multivariable model, hormonal secretion pattern remained independently associated with 30-day postoperative complications (*p* = 0.004), with hypercortisolism significantly associated with the outcome compared to non-functioning lesions (OR 2.413, 95% CI 1.319–4.415; *p* = 0.004). ASA score showed a borderline overall association (*p* = 0.066), although ASA score 3 was significantly associated with the outcome compared with ASA score 1 (OR 1.972, 95% CI 1.074–3.620; *p* = 0.028). Operative time (OR 1.008, 95% CI 1.003–1.014; *p* = 0.003), conversion to open surgery (OR 3.171, 95% CI 1.121–8.974; *p* = 0.030), and multiorgan resection (OR 3.698, 95% CI 1.178–11.609; *p* = 0.025) were also independently associated with 30-day postoperative complications.

The final multivariable model retained six predictors: ASA score, hormonal secretion pattern, operative time, conversion, ICU stay, and multiorgan resection. These predictors corresponded to nine estimated regression coefficients after accounting for the categorical coding of ASA score and hormonal secretion pattern. With 70 postoperative complication events, the final model therefore had approximately 7.8 events per estimated regression coefficient.

Linearity in the logit was satisfied for operative time, the only continuous predictor retained in the final multivariable model, using the Box–Tidwell approach (*p* = 0.248). The final model showed acceptable apparent discrimination for 30-day postoperative complications, with an AUC of 0.754 (95% CI 0.691–0.817; *p* < 0.001; Figure S1, Supplementary Materials). Internal validation using 1,000 bootstrap resamples yielded an optimism-corrected AUC of 0.730, indicating modest optimism in the apparent model performance. The Hosmer–Lemeshow goodness-of-fit test showed no evidence of lack of fit (χ^2^ = 1.287, 8 degrees of freedom; *p* = 0.996).

To explore the cumulative risk profile derived from this model, patients were stratified into tertiles according to their predicted probability of 30-day postoperative complications. Observed complication rates increased progressively across predicted-risk tertiles: 7 (2.6%) of 266 patients in the low-risk tertile, 14 (5.3%) of 266 in the intermediate-risk tertile, and 49 (18.4%) of 266 in the high-risk tertile (*p* for trend < 0.001; Figure S2, Supplementary Materials).

Among the 236 patients with hypercortisolism, 80 were classified as MACS and 156 as having overt Cushing syndrome. As detailed in Table S4 (Supplementary Materials), the incidence of postoperative complications was comparable between the two groups (12.5% vs 14.1%; OR 1.15, 95% CI 0.52–2.56; *p* = 0.734). Likewise, no significant differences were observed in Clavien–Dindo grade ≥ II complications or conversion rates.

### Secondary analyses

Exploratory approach-specific univariable and multivariable logistic regression analyses are shown for laparoscopic lateral transabdominal adrenalectomy, laparoscopic posterior retroperitoneoscopic adrenalectomy, and robotic lateral transabdominal adrenalectomy in Tables S6, S7, and S8, respectively. In patients undergoing laparoscopic lateral transabdominal adrenalectomy, ASA score was associated with 30-day postoperative complications as an overall variable at univariable analysis (*p* = 0.045), although no individual ASA category was significantly associated with the outcome compared with ASA score 1. Age (OR 1.035, 95% CI 1.004–1.066; *p* = 0.026), operative time (OR 1.013, 95% CI 1.007–1.019; *p* < 0.001), conversion to open surgery (OR 8.646, 95% CI 2.626–28.467; *p* < 0.001), multiorgan resection (OR 14.522, 95% CI 4.339–48.596; *p* < 0.001), intraoperative blood transfusion (OR 25.111, 95% CI 2.206–285.874; *p* = 0.009), and intraoperative complications (OR 5.489, 95% CI 1.340–22.486; *p* = 0.018) were also associated with the outcome. In the multivariable model, age (OR 1.036, 95% CI 1.004–1.070; *p* = 0.026), operative time (OR 1.010, 95% CI 1.002–1.017; *p* = 0.009), and multiorgan resection (OR 5.078, 95% CI 1.119–23.050; *p* = 0.035) remained independently associated with 30-day postoperative complications.

In patients undergoing laparoscopic posterior retroperitoneoscopic adrenalectomy, hormonal secretion pattern was associated with 30-day postoperative complications at univariable analysis (*p* = 0.048), with hypercortisolism showing a significant category-level association compared with non-functioning lesions (OR 4.808, 95% CI 1.287–17.933; *p* = 0.020). Age (OR 0.966, 95% CI 0.934–1.000; *p* = 0.048), operative time (OR 1.013, 95% CI 1.004–1.023; *p* = 0.004), bilateral adrenalectomy (OR 6.458, 95% CI 2.192–19.032; *p* < 0.001), and intensive care unit stay (OR 8.265, 95% CI 2.319–29.458; *p* < 0.001) were also associated with the outcome. In the multivariable model, operative time was the only variable independently associated with 30-day postoperative complications (OR 1.011, 95% CI 1.002–1.021; *p* = 0.022).

In patients undergoing robotic lateral transabdominal adrenalectomy, operative time (OR 1.012, 95% CI 1.001–1.022; *p* = 0.027), conversion to open surgery (OR 8.647, 95% CI 1.787–41.849; *p* = 0.007), and intensive care unit stay (OR 5.706, 95% CI 1.309–24.863; *p* = 0.020) were associated with 30-day postoperative complications at univariable analysis. In the multivariable model, conversion to open surgery was the only independent factor associated with the outcome (OR 6.171, 95% CI 1.101–34.579; *p* = 0.038).

Several subgroup estimates were characterised by wide confidence intervals or were not estimable because of sparse events within individual categories. These results should therefore be interpreted as exploratory and primarily according to the direction and consistency of the observed associations rather than the precise magnitude of the ORs.

### Other analyses

Table S9 shows the univariable and multivariable logistic regression analyses restricted to unilateral adrenalectomies. At univariable analysis, ASA score was associated with 30-day postoperative complications as an overall variable (*p* = 0.007), although no individual ASA category reached statistical significance. Previous abdominal surgery (OR 1.744, 95% CI 1.035–2.937; *p* = 0.037), operative time (OR 1.012, 95% CI 1.007–1.017; *p* < 0.001), conversion to open surgery (OR 7.302, 95% CI 3.080–17.310; *p* < 0.001), lymph node dissection (OR 2.526, 95% CI 1.124–5.677; *p* = 0.025), multiorgan resection (OR 9.333, 95% CI 3.603–24.179; *p* < 0.001), intraoperative blood transfusion (OR 35.746, 95% CI 3.661–349.024; *p* = 0.002), and intensive care unit stay (OR 5.264, 95% CI 2.308–12.004; *p* < 0.001) were associated with the outcome. The side of surgery was not significantly associated with 30-day postoperative complications at univariable analysis, although a borderline trend was observed for left-sided adrenalectomy compared with right-sided adrenalectomy (OR 1.648, 95% CI 0.968–2.805; *p* = 0.066). Pathological diagnosis was not significantly associated with postoperative complications as an overall variable (*p* = 0.229).

In the multivariable model restricted to unilateral adrenalectomies, surgical side was not independently associated with 30-day postoperative complications (left versus right: OR 1.334, 95% CI 0.755–2.358; *p* = 0.321). The overall pattern of hormonal hypersecretion had a borderline association with postoperative complications (*p* = 0.058), while hypercortisolism remained significantly associated with the outcome compared with non-functioning lesions (OR 2.047, 95% CI 1.079–3.884; *p* = 0.028). Operative time (OR 1.009, 95% CI 1.002–1.015; *p* = 0.006), conversion to open surgery (OR 3.145, 95% CI 1.096–9.030; *p* = 0.033), and multiorgan resection (OR 3.738, 95% CI 1.181–11.838; *p* = 0.025) were also independently associated with 30-day postoperative complications.

Temporal analysis showed no significant linear decline in 30-day postoperative complications across the overall series (coefficient for case order −0.00007; *p* = 0.105). However, CUSUM analysis suggested a time-dependent change in the complication pattern, and structural break testing identified a potential inflection point around the 221st case, corresponding to early 2018 (Supremum Wald test, *p* = 0.040). Thereafter, the trend appeared more stable over time (Figures S3 and S4, Supplementary Materials). Sensitivity analyses were subsequently performed by adding surgical era or chronological case order to the final multivariable model (Table S10, Supplementary Materials). Neither the later surgical era, defined as procedures performed from 2019 onwards versus those performed up to and including 2018 (OR 0.626, 95% CI 0.359–1.092; *p* = 0.099), nor chronological case order entered as a continuous variable (OR 0.999 per consecutive procedure, 95% CI 0.998–1.000; *p* = 0.084) was independently associated with 30-day postoperative complications. Adjustment for either temporal covariate did not materially alter the direction or magnitude of the principal associations identified in the primary model. ASA score 3, hypercortisolism, operative time, conversion to open surgery, and multiorgan resection remained independently associated with postoperative complications in both sensitivity analyses.

## Discussion

This single-centre retrospective cohort study suggests that early postoperative morbidity after minimally invasive adrenalectomy is primarily associated with baseline patient status, hormonal secretion pattern, and procedural complexity rather than with the minimally invasive access route alone. Among 798 patients, 30-day postoperative complications occurred in 8.8%, and ASA score 3, hypercortisolism, longer operative time, conversion to open surgery, and multiorgan resection were independently associated with this outcome. Surgical approach and laterality were not independently associated with postoperative morbidity.

The multivariable model was developed to characterise these associations rather than to provide individualised clinical prediction. It showed acceptable apparent discrimination, with an AUC of 0.754, which decreased modestly to 0.730 after bootstrap optimism correction. The Hosmer–Lemeshow test showed no evidence of poor fit. These findings support the internal consistency of the model but should not be interpreted as validation of a clinical risk-prediction tool.

Minimally invasive adrenalectomy has substantially reduced surgical trauma and is now the standard approach for most benign adrenal lesions, as well as for selected malignant and high-risk endocrine conditions when oncological and technical principles can be respected [1, 3, 6, 8, 36–38]. Nevertheless, postoperative complications remain clinically relevant, particularly in patients with substantial comorbidity, hormone-secreting disease, complex lesions, or adverse intraoperative events [24–26, 39]. The 8.8% 30-day complication rate observed in the present series is consistent with previous large institutional and registry-based studies. Chen et al. [24] described Clavien–Dindo grade ≥ 2 complications in 8.4% of patients undergoing laparoscopic adrenalectomy, Van Den Heede et al. [40] reported grade ≥2 morbidity in 3.6% of 2,652 patients included in the EUROCRINE® registry, and Fouché et al. [26] indicated postoperative complications in 13.4% of lateral transperitoneal laparoscopic adrenalectomies. Differences among studies probably reflect heterogeneity in complication definitions, case mix, hormonal secretion patterns, operative approaches, procedural complexity, surgeon experience, and institutional volume [41, 42].

The independent association between ASA score 3 and postoperative complications supports the role of impaired preoperative physical status and reduced physiological reserve in determining early morbidity. Although the ASA score is less granular than dedicated comorbidity or frailty indices, it remains widely available in routine practice and provides a pragmatic summary of baseline risk. This finding is consistent with previous adrenalectomy series: Chen et al. [24] identified ASA class 3–4 as independently associated with complications after laparoscopic adrenalectomy, Hattori et al. [43] reported ASA physical status grade ≥3 as the only independent predictor of perioperative complications, and Parente et al. [44] found that an age-adjusted Charlson Comorbidity Index ≥ 3 was independently associated with postoperative complications after adrenalectomy for phaeochromocytoma.

The association between hypercortisolism and postoperative morbidity is biologically plausible because chronic cortisol excess adversely affects cardiovascular, metabolic, immune, thrombotic, musculoskeletal, and tissue-repair pathways [31, 32, 42]. This finding is also consistent with previous surgical series. In a propensity score-matched analysis from our group, hypercortisolism was independently associated with postoperative morbidity after lateral transabdominal adrenalectomy [34], while Prudhomme et al. [45] identified Cushing’s syndrome as the only factor associated with major postoperative complications at univariable analysis. More broadly, active Cushing’s syndrome is associated with a substantial burden of infectious, thromboembolic, metabolic, electrolyte-related, and cardiovascular complications [46].

In the present cohort, patients with hypercortisolism had approximately twice the likelihood of developing postoperative complications compared with patients with other hormonal secretion patterns, both for all Clavien–Dindo grades and for grade ≥II events. Surgical or procedure-related complications were significantly more frequent in patients with hypercortisolism, whereas the numerical increase in medical complications did not reach statistical significance. These findings suggest that cortisol excess may confer a broad perioperative vulnerability rather than a predisposition to a single complication category. However, the relatively small numbers of medical and surgical events limited the ability to distinguish specific complication patterns. Postoperative complication rates were also comparable between patients with MACS and overt Cushing syndrome. This exploratory comparison included only 32 events among 236 patients with hypercortisolism and was not powered to determine whether the severity of cortisol excess independently modifies postoperative risk. The absence of a significant difference should therefore not be interpreted as evidence of equivalent perioperative risk, but rather as an indication that the present study could not reliably distinguish the respective effects of MACS and overt Cushing syndrome.

Longer operative time, conversion to open surgery, and multiorgan resection collectively identify procedural complexity as an important component of postoperative risk. Operative time remained independently associated with complications, consistent with our previous propensity score-matched analysis, in which an operative duration > 75 min was associated with postoperative morbidity [34]. Other studies have similarly linked prolonged or technically demanding procedures to adverse outcomes. Tiberio et al. [47] reported that operative duration > 140 min was associated with intraoperative complications and prolonged hospital stay, whereas Bittner et al. [48] identified operative time > 180 min, conversion, concomitant procedures, blood loss > 200 mL, large indeterminate masses, adrenocortical carcinoma, and tumour size > 6 cm as predictors of 30-day morbidity.

Conversion to open surgery should not be regarded as a preoperative risk factor or as a direct cause of morbidity, but rather as a marker of technical difficulty, unexpected intraoperative findings, or the need to escalate the operative strategy. This interpretation is consistent with Gaujoux et al. [39], who identified conversion as an independent predictor of complications after unilateral laparoscopic adrenalectomy, and with Thompson et al. [25] who reported conversion as the only independently associated factor in a national adrenalectomy registry.

Multiorgan resection should be interpreted similarly as a marker of greater procedural complexity. In the present cohort, additional organ resections were undertaken based on preoperative imaging or intraoperative assessment. When adjacent-organ involvement was suspected, histopathological invasion was confirmed only in a subset of patients, reflecting the need for operative decisions to precede definitive histopathology. In suspected or locally advanced adrenal malignancy, en bloc resection of involved structures may be required to achieve complete tumour clearance and avoid capsular rupture, whereas routine resection of uninvolved organs is not supported [49, 50]. Such procedures should therefore be concentrated in high-volume referral centres with multidisciplinary expertise.

The approach-specific analyses were exploratory because of the limited number of events within individual subgroups. Age, operative time, and multiorgan resection remained independently associated with complications after laparoscopic lateral transabdominal adrenalectomy; operative time was the only independently associated factor after laparoscopic posterior retroperitoneoscopic adrenalectomy; and conversion was the only independently associated factor after robotic lateral transabdominal adrenalectomy. Although these findings are broadly consistent with previous reports linking age, operative difficulty, concomitant procedures, and conversion to perioperative morbidity [26, 48, 51], sparse events and wide confidence intervals preclude firm approach-specific conclusions. Rather than indicating superiority or inferiority of a particular technique, these analyses reinforce the importance of patient selection and procedural complexity.

Surgical laterality was not independently associated with 30-day postoperative complications in the analysis restricted to unilateral adrenalectomies, despite previous reports of side-specific differences in morbidity or technical difficulty [26, 39, 52]. Temporal analysis also showed no significant linear decline in complications across the study period. Although CUSUM analysis identified a potential inflection point around the 221st case, corresponding to early 2018, sensitivity analyses adjusted for either surgical era or chronological case order did not materially alter the principal associations. Neither temporal covariate was independently associated with postoperative morbidity. These findings suggest that the main results were not substantially explained by changes in institutional experience, technology, or perioperative practice. Because procedures were performed by experienced endocrine surgeons and, from 2017 onwards, were performed or supervised by the same expert surgeon, the CUSUM pattern is more appropriately interpreted as temporal stabilisation and procedural standardisation than as a formal learning-curve effect.

Taken together, these findings support an integrated interpretation of postoperative risk rather than the evaluation of individual factors in isolation. The model showed acceptable discrimination and observed complication rates increased from 2.6% in the lowest predicted-risk tertile to 18.4% in the highest. This gradient indicates that morbidity was more frequent when adverse clinical, endocrine, and operative features coexisted. However, the tertiles were used descriptively and should not be regarded as validated clinical risk categories, while the model itself was not developed for individual preoperative prediction.

This interpretation is consistent with previous studies of operative complexity and postoperative risk. Alberici et al. [53] developed a preoperative difficulty score for laparoscopic transabdominal adrenalectomy incorporating patient characteristics, lesion features, surgical experience, extended resection, and associated procedures. Similarly, Parente et al. [44] identified comorbidity burden and conversion or open surgery as independently associated with postoperative complications after adrenalectomy for phaeochromocytoma.

This study has several strengths. It included a large consecutive cohort of minimally invasive adrenalectomies performed at a high-volume endocrine surgery referral centre with long-standing experience in adrenal surgery. The single-centre design reduced heterogeneity in surgical reporting, perioperative management, and complication assessment. Standardised 30-day outcome definitions, Clavien–Dindo grading, detailed clinical, endocrine, operative, and histopathological variables, and multivariable modelling with assessment of discrimination, calibration, and internal validation further support the robustness of the analysis. Moreover, the learning curves for laparoscopic lateral transabdominal and posterior retroperitoneoscopic adrenalectomy had been completed before the study period.

These findings should be interpreted in the light of several limitations. First, the retrospective design may have introduced selection, information, and residual confounding and precludes causal inference. Second, the single-centre setting may limit generalisability to institutions with different operative volumes, expertise, patient selection, and perioperative pathways. Third, the approximately 14-year study period encompassed changes in case volume, perioperative care, surgical approach distribution, and robotic platforms. Robotic adrenalectomy was introduced during the study period, several platforms were progressively adopted, and robotic posterior retroperitoneoscopic adrenalectomy was introduced only in 2025. Although adjustment for surgical era and chronological case order did not materially alter the principal associations, residual temporal and platform-related confounding cannot be excluded.

Fourth, ASA score was used as a pragmatic measure of preoperative physical status, whereas more granular measures of comorbidity, frailty, sarcopenia, and nutritional status were unavailable. Hypercortisolism was analysed as a hormonal secretion pattern, and the limited number of events among patients with MACS and overt Cushing syndrome precluded reliable multivariable assessment of cortisol-excess severity. Disease duration, preoperative hormonal control, and individual cortisol-related comorbidities were also not incorporated.

Fifth, approach-specific analyses were limited by small subgroup sizes, sparse events, and imprecise estimates and should therefore be considered exploratory. Finally, the primary model incorporated intraoperative and perioperative variables, including operative time, conversion, ICU stay, and multiorgan resection, which are not fully available before surgery. It was therefore not designed as a preoperative risk score. Bootstrap validation showed only modest optimism and the Hosmer–Lemeshow test showed no evidence of poor fit, but both were based on the same cohort used for model development. Any predictive application would require a separately developed model restricted to preoperatively available variables and external validation in independent cohorts.

In conclusion, ASA score 3, hypercortisolism, longer operative time, conversion to open surgery, and multiorgan resection were independently associated with 30-day postoperative complications after minimally invasive adrenalectomy, whereas surgical approach and laterality were not. These findings support a multidimensional interpretation of postoperative morbidity integrating baseline patient status, hormonal secretion pattern, and procedural complexity rather than relying on the minimally invasive access route alone. The model should be interpreted as explanatory and associational, not as a dedicated preoperative prediction tool. Further multicentre studies are required to confirm these associations and to develop and externally validate models specifically intended for preoperative risk estimation.

## Supplementary Information

Below is the link to the electronic supplementary material.

Supplementary file1 (DOCX 65 KB)

Supplementary file2 (TIF 51 KB)

Supplementary file3 (TIF 48 KB)

Supplementary file4 (TIF 2621 KB)

Supplementary file5 (TIF 2621 KB)

## Data Availability

The datasets analysed during the current study are available from the corresponding author on reasonable request.

## Declarations

### Research involving human and animal participants

This study involved human participants and did not include animal experiments.

### Study protocol

No separate study protocol was published or registered.

### Artificial intelligence assistance

ChatGPT (OpenAI, San Francisco, CA, USA) was used solely to support English-language editing and stylistic refinement during manuscript preparation and revision. The tool was not used for study design, data collection, statistical analysis, interpretation of the results, or reference generation. Only manuscript text and anonymised or aggregated information were provided; no identifiable patient data were entered. All AI-assisted outputs were critically reviewed, verified, and edited by the authors, who take full responsibility for the accuracy, integrity, and final content of the manuscript.

### Patient and public involvement

Patients and members of the public were not involved in the design, conduct, reporting, or dissemination of this research.
