# Supplementary material for: Risk factors for early postoperative complications after minimally invasive adrenalectomy: a single-centre retrospective cohort study of more than 800 patients
Source: Surg Endosc. 2026 Aug 31;40(10):9122–41. doi: 10.1007/s00464-026-13308-0 (PMC13633292; doi:10.1007/s00464-026-13308-0)
Supplement: Supplementary file 1 [file 464_2026_13308_MOESM1_ESM.docx]

**Supplementary materials**

**Table S1.** Distribution of robotic adrenalectomy procedures according to robotic platform and surgical approach

| **Robotic platforms** | LTA | PRA |
| --- | --- | --- |
| **Da Vinci Si** | 68 | - |
| **Da Vinci Xi** | 121 | 14 |
| **HUGO™-RAS** | 17 | - |
| **Versius®** | 10 | - |
| **Toumai®** | 4 | 7 |

LTA, Lateral Transabdominal Adrenalectomy; PRA, Posterior Retroperitoneoscopic Adrenalectomy

**Table S2.** Comparison of preoperative, intraoperative, postoperative, and histopathological characteristics according to surgical procedure

|  | L-LTA | L-PRA | R-LTA | R-PRA |
| --- | --- | --- | --- | --- |
| **Patients** | 369 | 188 | 220 | 21 |
| **Age (years)** | 57.0 (48.0-67.0) | 54.5 (45.0-64.0) | 56.0 (46.0-65.0) | 61.0 (50.0-67.5) |
| **Sex***  **Male**  **Female** | 135 (36.6%)  234 (63.4%) | 64 (34.0%)  124 (66.0%) | 79 (35.9%)  141 (64.1%) | 6 (28.6%)  15 (71.4%) |
| **BMI (kg/m^2^)** | 25.7 (23.1-29.2) | 24.2 (22.1-27.0) | 26.3 (22.8-31.2) | 25.3 (22.7-30.2) |
| **ASA score**  **1**  **2**  **3** | 11 (3.0%)  265 (71.8%)  93 (25.2%) | 3 (1.6%)  159 (84.6%)  26 (13.8%) | 8 (3.6%)  170 (77.3%)  42 (19.1%) | 0 (0%)  16 (76.2%)  5 (23.8%) |
| **Pattern of hormonal hypersecretion**  **Non-functioning**  **Primary hyperaldosteronism**  **Catecholamine hypersecretion**  **Hypercortisolism** | 184 (49.9%)  20 (5.4%)  62 (16.8%)  103 (27.9%) | 54 (28.7%)  30 (16.0%)  45 (23.9%)  59 (31.4%) | 101 (45.9%)  11 (5.0%)  44 (20.0%)  64 (29.1%) | 5 (23.8%)  4 (19.0%)  2 (9.5%)  10 (47.7%) |
| **Previous abdominal surgery**  **No**  **Yes** | 219 (59.3%)  150 (40.7%) | 101 (53.7%)  87 (46.3%) | 140 (63.6%)  80 (36.4%) | 15 (71.4%)  6 (28.6%) |
| **Operative time (minutes)**  **Docking time (minutes)**  **Console time (minutes)** | 65.0 (46.5-100.0)  -  - | 55.0 (40.0-90.0)  -  - | 90.0 (70.0-120.0)  7.0 (6.0-8.0)  37.0 (26.5-51.0) | 58.0 (45.5-75.0)  7.0 (5.0-10.0)  17.0 (12.0-24.0) |
| **Conversion**  **No**  **Yes** | 356 (96.5%)  13 (3.5%) | 183 (97.3%)  5 (2.7%) | 213 (96.8%)  7 (3.2%) | 21 (100%)  0 (0%) |
| **Laterality**  **Right**  **Left**  **Bilateral** | 185 (50.1%)  174 (47.2%)  10 (2.7%) | 73 (38.8%)  94 (50.0%)  21 (11.2%) | 117 (53.2%)  102 (46.4%)  1 (0.4%) | 8 (38.1%)  13 (61.9%)  0 (0%) |
| **Extent of adrenal surgery**  **Unilateral**  **Bilateral** | 359 (97.3%)  10 (2.7%) | 167 (88.8%)  21 (11.2%) | 219 (99.5%)  1 (0.5%) | 21 (100%)  0 (0%) |
| **Partial adrenalectomy**  **No**  **Yes** | 362 (98.1%)  7 (1.9%) | 184 (97.9%)  4 (2.1%) | 219 (99.5%)  1 (0.5%) | 21 (100%)  0 (0%) |
| **Lymph node dissection**  **No**  **Yes** | 337 (91.3%)  32 (8.7%) | 185 (98.4%)  3 (1.6%) | 208 (94.5%)  12 (5.5%) | 21 (100%)  0 (0%) |
| **Multiorgan resection**  **No**  **Yes** | 357 (96.7%)  12 (3.3%) | 185 (98.4%)  3 (1.6%) | 216 (98.2%)  4 (1.8%) | 21 (100%)  0 (0%) |
| **Intraoperative blood transfusion**  **No**  **Yes** | 366 (99.2%)  3 (0.8%) | 188 (100%)  0 (0%) | 219 (99.5%)  1 (0.5%) | 21 (100%)  0 (0%) |
| **Intraoperative complications**  **No**  **Yes** | 359 (97.3%)  10 (2.7%) | 183 (97.3%)  5 (2.7%) | 215 (97.7%)  5 (2.3%) | 20 (95.2%)  1 (4.8%) |
| **ICU stay**  **No**  **Yes** | 347 (94.0%)  22 (6.0%) | 176 (93.6%)  12 (6.4%) | 211 (95.9%)  9 (4.1%) | 21 (100%)  0 (0%) |
| **30-day postoperative complications**  **No**  **Yes** | 340 (92.1%)  29 (7.9%) | 169 (89.9%)  19 (10.1%) | 200 (90.9%)  20 (9.1%) | 19 (90.5%)  2 (9.5%) |
| **Clavien-Dindo grade**  **0**  **I**  **II**  **IIIa**  **IIIb**  **IV**  **V** | 340 (92.1%)  4 (1.1%)  19 (5.2%)  0 (0%)  2 (0.5%)  4 (1.1%)  0 (0%) | 169 (89.9%)  2 (1.1%)  12 (6.4%)  2 (1.1%)  0 (0%)  2 (1.0%)  1 (0.5%) | 200 (90.9%)  7 (3.2%)  11 (4.9%)  0 (0%)  1 (0.5%)  1 (0.5%)  0 (0%) | 19 (90.5%)  1 (4.75%)  0 (0%)  0 (0%)  0 (0%)  0 (0%)  1 (4.75%) |
| **Mortality within 6 months from the index operation** | 1 (0.3%) | 2 (1.1%) | 0 (0%) | 1 (4.8%) |
| **Postoperative hospital stay (days)** | 3.0 (2.0-4.0) | 3.0 (2.0-4.0) | 3.0 (2.0-4.0) | 2.0 (2.0-2.0) |
| **Lesion size (cm)** | 4.5 (3.2-6.8) | 3.0 (2.3-4.5) | 4.8 (3.5-6.0) | 3.0 (2.7-4.7) |
| **Pathological diagnosis**  **Benign lesions**  **Pheochromocytomas**  **Adrenocortical neoplasms of uncertain malignant potential**  **Adrenocortical carcinomas**  **Adrenal metastases**  **Other malignancies** | 260 (70.5%)  71 (19.2%)  7 (1.9%)  16 (4.3%)  12 (3.3%)  3 (0.8%) | 118 (62.8%)  50 (26.6%)  3 (1.6%)  1 (0.5%)  15 (8.0%)  1 (0.5%) | 151 (68.7%)  46 (20.9%)  5 (2.3%)  8 (3.6%)  8 (3.6%)  2 (0.9%) | 17 (81.0%)  2 (9.5%)  0 (0%)  0 (0%)  2 (9.5%)  0 (0%) |

L, Laparoscopic; R, Robotic; LTA, Lateral Transabdominal Adrenalectomy; PRA, Posterior Retroperitoneoscopic Adrenalectomy; ICU, Intensive Care Unit.

All continuous variables are reported as median and interquartile range (Q1–Q3), whereas categorical variables are reported as number and percentage.

* Sex as assigned at birth.

**Table S3.** Preoperative, intraoperative, postoperative, and histopathological characteristics of patients undergoing unilateral adrenalectomy

| **Patients** | 766 |
| --- | --- |
| **Age (years)** | 56.5 (47.0-66.0) |
| **Sex***  **Male**  **Female** | 272 (35.6%)  494 (64.4%) |
| **BMI (kg/m^2^)** | 25.5 (22.8-29.3) |
| **ASA score**  **1**  **2**  **3** | 21 (2.7%)  583 (76.1%)  162 (21.2%) |
| **Hormonal hypersecretion**  **No**  **Yes** | 344 (44.9%)  422 (55.1%) |
| **Pattern of hormonal hypersecretion**  **Non-functioning**  **Primary hyperaldosteronism**  **Catecholamine hypersecretion**  **Hypercortisolism** | 344 (44.9%)  65 (8.5%)  143 (18.7%)  214 (27.9%) |
| **Previous abdominal surgery**  **No**  **Yes** | 455 (59.4%)  311 (40.6%) |
| **Operative time (minutes)** | 70 (49-100) |
| **Surgical procedure**  **Laparoscopic LTA**  **Laparoscopic PRA**  **Robotic LTA**  **Robotic PRA** | 359 (46.9%)  167 (21.8%)  219 (28.6%)  21 (2.7%) |
| **Conversion**  **No**  **Yes** | 741 (96.7%)  25 (3.3%) |
| **Laterality**  **Right**  **Left** | 383 (50.0%)  383 (50.0%) |
| **Partial adrenalectomy**  **No**  **Yes** | 758 (99.0%)  8 (1.0%) |
| **Lymph node dissection**  **No**  **Yes** | 719 (93.9%)  47 (6.1%) |
| **Multiorgan resection**  **No**  **Yes** | 747 (97.5%)  19 (2.5%) |
| **Intraoperative blood transfusion**  **No**  **Yes** | 762 (99.5%)  4 (0.5%) |
| **Intraoperative complications**  **No**  **Yes** | 745 (97.3%)  21 (2.7%) |
| **ICU stay**  **No**  **Yes** | 735 (96.0%)  31 (4.0%) |
| **30-day postoperative complications**  **No**  **Yes** | 704 (91.9%)  62 (8.1%) |
| **Clavien-Dindo grade**  **0**  **I**  **II**  **IIIa**  **IIIb**  **IV**  **V** | 704 (91.9%)  14 (1.8%)  37 (4.9%)  0 (0%)  3 (0.4%)  7 (0.9%)  1 (0.1%) |
| **Mortality within 6 months from the index operation** | 2 (0.3%) |
| **Postoperative hospital stay (days)** | 3.0 (2.0-4.0) |
| **Lesion size (cm)** | 4.0 (3.0-6.0) |
| **Pathological diagnosis**  **Benign lesions**  **Pheochromocytomas**  **Adrenocortical neoplasms of uncertain malignant potential**  **Adrenocortical carcinomas**  **Adrenal metastases**  **Other malignancies** | 524 (68.4%)  159 (20.8%)  15 (1.9%)  25 (3.3%)  37 (4.8%)  6 (0.8%) |

LTA, Lateral Transabdominal Adrenalectomy; PRA, Posterior Retroperitoneoscopic Adrenalectomy; ICU, Intensive Care Unit.

All continuous variables are reported as median and interquartile range (Q1–Q3), whereas categorical variables are reported as number and percentage.

* Sex as assigned at birth.

**Table S4.** Comparison of demographic, clinical, operative, and postoperative characteristics between patients with mild autonomous cortisol secretion and overt Cushing syndrome

|  | **MACS** | **Overt Cushing syndrome** | **p-value** |
| --- | --- | --- | --- |
| **Patients** | 80 | 156 | - |
| **Age (years)** | 61.5 (51.3-68.0) | 55.0 (46.0-65.0) | **0.022** |
| **Sex***  **Male**  **Female** | 19 (23.8%) 61 (76.2%) | 32 (20.5%) 124 (79.5%) | 0.567 |
| **BMI (kg/m²)** | 23.7 (22.3-25.4) | 29.9 (26.7-33.2) | **<0.001** |
| **ASA score**  **1**  **2**  **3** | 1 (1.2%) 65 (81.3%) 14 (17.5%) | 3 (1.9%) 113 (72.5%) 40 (25.6%) | 0.330 |
| **Surgical procedure**  **Laparoscopic LTA**  **Laparoscopic PRA**  **Robotic LTA**  **Robotic PRA** | 35 (43.8%) 26 (32.5%) 14 (17.5%) 5 (6.2%) | 68 (43.6%) 33 (21.2%) 50 (32.1%) 5 (3.1%) | **0.046** |
| **Operative time (minutes)** | 56.5 (43.3-90.0) | 75.0 (54.5-105.3) | **0.009** |
| **Conversion**  **No**  **Yes** | 78 (97.5%) 2 (2.5%) | 155 (99.4%) 1 (0.6%) | 0.266 |
| **30-day postoperative complications**  **No**  **Yes** | 70 (87.5%) 10 (12.5%) | 134 (85.9%) 22 (14.1%) | 0.734 |
| **Clavien-Dindo grade ≥II**  **No**  **Yes** | 72 (90%) 8 (10.0%) | 138 (88.5%) 18 (11.5%) | 0.828 |
| **Postoperative hospital stay (days)** | 3.0 (2.0-5.0) | 4.0 (2.0-5.0) | **0.019** |
| **Lesion size (cm)** | 3.6 (2.7-4.4) | 4.0 (3.0-5.0) | 0.174 |

LTA, Lateral Transabdominal Adrenalectomy; PRA, Posterior Retroperitoneoscopic Adrenalectomy; MACS, mild autonomous cortisol secretion.

All continuous variables are reported as median and interquartile range (Q1–Q3), whereas categorical variables are reported as number and percentage. Bold p-values indicate statistical significance at p<0.05.

* Sex as assigned at birth.

**Table S5.** Distribution of 30-day postoperative complications according to hypercortisolism and complication type

| **Outcome** | **Hypercortisolism (n = 236)** | **Other hormonal secretion patterns (n = 562)** | **OR (95% CI)** | **p-value** |
| --- | --- | --- | --- | --- |
| **Any complication, Clavien-Dindo grade ≥I** | 32 (13.6%) | 38 (6.8%) | 2.16 (1.32-3.56) | **0.004** |
| **Medical complication, Clavien-Dindo grade ≥I** | 14 (5.9%) | 22 (3.9%) | 1.55 (0.78-3.08) | 0.261 |
| **Surgical/procedure-related complication, Clavien-Dindo grade ≥I** | 18 (7.6%) | 16 (2.8%) | 2.82 (1.41-5.63) | **0.004** |
| **Any complication, Clavien-Dindo grade ≥II** | 26 (11.0%) | 30 (5.3%) | 2.20 (1.27-3.80) | **0.006** |
| **Medical complication, Clavien-Dindo grade ≥II** | 13 (5.5%) | 18 (3.2%) | 1.76 (0.85-3.66) | 0.158 |
| **Surgical/procedure-related complication, Clavien-Dindo grade ≥II** | 13 (5.5%) | 12 (2.1%) | 2.67 (1.20-5.95) | **0.023** |

Values are presented as number (percentage). Odds ratios compare patients with hypercortisolism with those with other hormonal secretion patterns. Medical and surgical/procedure-related complications were classified according to the presumed primary nature of the postoperative event. Clavien–Dindo grade ≥I includes all recorded postoperative complications, whereas grade ≥II identifies clinically relevant complications requiring pharmacological, interventional, surgical, or intensive care treatment.

Bold p-values indicate statistical significance at p<0.05.

**Table S6.** Univariable and multivariable logistic regression analyses of factors associated with 30-day postoperative complications in patients undergoing laparoscopic lateral transabdominal adrenalectomy

|  | **Univariable analysis** | **Multivariable analysis** |
| --- | --- | --- |
| **Age (years)** | **1.035 (1.004-1.066)**  **p=0.026** | **1.036 (1.004-1.070)**  **p=0.026** |
| **Sex***  **Male (reference)**  **Female** | 0.939 (0.430-2.053)  p=0.875 |  |
| **BMI (kg/m^2^)** | 0.987 (0.919-1.059)  p=0.714 |  |
| **ASA score**  **1 (reference)**  **2**  **3** | **p=0.045**  1.410 (0.311-6.390)  p=0.656  1.850 (0.701-4.885)  p=0.214 | p=0.201  0.556 (0.108-2.872)  p=0.483  2.314 (0.844-6.345)  p=0.103 |
| **Pattern of hormonal hypersecretion**  **Non-functioning (reference)**  **Primary hyperaldosteronism**  **Catecholamine hypersecretion**  **Hypercortisolism** | p=0.857  0.730 (0.692-5.589)  p=0.730  1.409 (0.512-3.882)  p=0.507  1.259 (0.519-3.056)  p=0.610 |  |
| **Previous abdominal surgery**  **No (reference)**  **Yes** | 1.204 (0.561-2.583)  p=0.634 |  |
| **Operative time (minutes)** | **1.013 (1.007-1.019)**  **p<0.001** | **1.010 (1.002-1.017)**  **p=0.009** |
| **Conversion**  **No (reference)**  **Yes** | **8.646 (2.626-28.467)**  **p<0.001** | 2.148 (0.477-9.672)  p=0.319 |
| **Extent of adrenal surgery**  **Unilateral (reference)**  **Bilateral** | 1.313 (0.161-10.744)  p=0.799 |  |
| **Partial adrenalectomy**  **No (reference)**  **Yes** | *Not estimable* |  |
| **Lymph node dissection**  **No (reference)**  **Yes** | **2.415 (0.853-6.837)**  **p=0.097** | 0.431 (0.089-2.085)  p=0.295 |
| **Multiorgan resection**  **No (reference)**  **Yes** | **14.522 (4.339-48.596)**  **p<0.001** | **5.078 (1.119-23.050)**  **p=0.035** |
| **Intraoperative blood transfusion**  **No (reference)**  **Yes** | **25.111 (2.206-285.874)**  **p=0.009** | 0.460 (0.019-11.082)  p=0.632 |
| **Intraoperative complications**  **No (reference)**  **Yes** | **5.489 (1.340-22.486)**  **p=0.018** | 0.472 (0.056-3.971)  p=0.489 |
| **ICU stay**  **No (reference)**  **Yes** | **2.862 (0.900-9.106)**  **p=0.075** | 1.708 (0.411-7.107)  p=0.643 |
| **Lesion size (cm)** | 0.997 (0.983-1.012)  p=0.719 |  |
| **Pathological diagnosis**  **Benign lesions (reference)**  **Pheochromocytomas**  **Adrenocortical neoplasms of uncertain malignant potential**  **Adrenocortical carcinomas**  **Adrenal metastases**  **Other malignancies** | p=0.249  1.937 (0.793-4.729)  p=0.147  6.100 (1.097-33.932)  p=0.039  1.017 (0.126-8.191)  p=0.988  3.050 (0.616-15.110)  p=0.172  *Not estimable* |  |

ICU, Intensive Care Unit

The values reported in the table are odds ratios, with 95% confidence intervals and corresponding p-values.

Variables not retained in the final multivariable model are left blank. In the univariable analysis, bold entries identify candidate variables meeting the predefined p<0.20 criterion for inclusion in the multivariable model; in the multivariable analysis, bold entries indicate statistically significant associations at p<0.05.

* Sex as assigned at birth.

**Table S7.** Univariable and multivariable logistic regression analyses of factors associated with 30-day postoperative complications in patients undergoing laparoscopic posterior retroperitoneoscopic adrenalectomy

|  | **Univariable analysis** | **Multivariable analysis** |
| --- | --- | --- |
| **Age (years)** | **0.966 (0.934-1.000)**  **p=0.048** | 0.967 (0.932-1.004)  p=0.077 |
| **Sex***  **Male (reference)**  **Female** | 0.681 (0.260-1.789)  p=0.436 |  |
| **BMI (kg/m^2^)** | **1.081 (0.972-1.202)**  **p=0.153** | 1.113 (0.981-1.262)  p=0.095 |
| **ASA score**  **1 (reference)**  **2**  **3** | p=0.446  0.387 (0.058-2.582)  p=0.327  2.080 (0.640-6.761)  p=0.224 |  |
| **Pattern of hormonal hypersecretion**  **Non-functioning (reference)**  **Primary hyperaldosteronism**  **Catecholamine hypersecretion**  **Hypercortisolism** | **p=0.048**  ***Not estimable***  **1.214 (0.233-6.333)**  **p=0.818**  **4.808 (1.287-17.933)**  **p=0.020** | p=0.067  *Not estimable*  0.873 (0.143-5.333)  p=0.883  4.013 (0.990-16.267)  p=0.052 |
| **Previous abdominal surgery**  **No (reference)**  **Yes** | 1.328 (0.513-3.433)  p=0.559 |  |
| **Operative time (minutes)** | **1.013 (1.004-1.023)**  **p=0.004** | **1.011 (1.002-1.021)**  **p=0.022** |
| **Conversion**  **No (reference)**  **Yes** | 2.292 (0.243-21.627)  p=0.469 |  |
| **Extent of adrenal surgery**  **Unilateral (reference)**  **Bilateral** | **6.458 (2.192-19.032)**  **p<0.001** | 1.059 (0.233-4.806)  p=0.941 |
| **Partial adrenalectomy**  **No (reference)**  **Yes** | *Not estimable* |  |
| **Lymph node dissection**  **No (reference)**  **Yes** | *Not estimable* |  |
| **Multiorgan resection**  **No (reference)**  **Yes** | 4.639 (0.401-53.714)  p=0.219 |  |
| **Intraoperative blood transfusion**  **No (reference)**  **Yes** | *Not estimable* |  |
| **Intraoperative complications**  **No (reference)**  **Yes** | *Not estimable* |  |
| **ICU stay**  **No (reference)**  **Yes** | **8.265 (2.319-29.458)**  **p<0.001** | 3.140 (0.687-14.349)  p=0.140 |
| **Lesion size (cm)** | 1.016 (0.989-1.043)  p=0.251 |  |
| **Pathological diagnosis**  **Benign lesions (reference)**  **Pheochromocytomas**  **Adrenocortical neoplasms of uncertain malignant potential**  **Adrenocortical carcinomas**  **Adrenal metastases**  **Other malignancies** | p=0.947  0.516 (0.140-1.895)  p=0.318  *Not estimable*  *Not estimable*  1.243 (0.252-6.132)  p=0.790  *Not estimable* |  |

The values reported in the table are odds ratios, with 95% confidence intervals and corresponding p-values.

Variables not retained in the final multivariable model are left blank. In the univariable analysis, bold entries identify candidate variables meeting the predefined p<0.20 criterion for inclusion in the multivariable model; in the multivariable analysis, bold entries indicate statistically significant associations at p<0.05.

* Sex as assigned at birth.

**Table S8.** Univariable and multivariable logistic regression analyses of factors associated with 30-day postoperative complications in patients undergoing robotic lateral transabdominal adrenalectomy

|  | **Univariable analysis** | **Multivariable analysis** |
| --- | --- | --- |
| **Age (years)** | 1.014 (0.978-1.051)  p=0.452 |  |
| **Sex***  **Male (reference)**  **Female** | 1.341 (0.494-3.641)  p=0.565 |  |
| **BMI (kg/m^2^)** | 1.047 (0.972-1.128)  p=0.222 |  |
| **ASA score**  **1 (reference)**  **2**  **3** | p=0.395  1.115 (0.225-5.538)  p=0.894  1.662 (0.591-4.676)  p=0.335 |  |
| **Pattern of hormonal hypersecretion**  **Non-functioning (reference)**  **Primary hyperaldosteronism**  **Catecholamine hypersecretion**  **Hypercortisolism** | p=0.612  *Not estimable*  0.433 (0.091-2.065)  p=0.294  1.300 (0.484-3.490)  p=0.603 |  |
| **Previous abdominal surgery**  **No (reference)**  **Yes** | **2.320 (0.917-5.869)**  **p=0.075** | 2.400 (0.915-6.295)  p=0.075 |
| **Operative time (minutes)** | **1.012 (1.001-1.022)**  **p=0.027** | 1.005 (0.992-1.018)  p=0.449 |
| **Conversion**  **No (reference)**  **Yes** | **8.647 (1.787-41.849)**  **p=0.007** | **6.171 (1.101-34.579)**  **p=0.038** |
| **Extent of adrenal surgery**  **Unilateral (reference)**  **Bilateral** | *Not estimable* |  |
| **Partial adrenalectomy**  **No (reference)**  **Yes** | *Not estimable* |  |
| **Lymph node dissection**  **No (reference)**  **Yes** | **3.745 (0.926-15.151)**  **p=0.064** | 2.907 (0.670-12.619)  p=0.154 |
| **Multiorgan resection**  **No (reference)**  **Yes** | 3.456 (0.343-34.875)  p=0.293 |  |
| **Intraoperative blood transfusion**  **No (reference)**  **Yes** | *Not estimable* |  |
| **Intraoperative complications**  **No (reference)**  **Yes** | 2.579 (0.274-24.257)  p=0.407 |  |
| **ICU stay**  **No (reference)**  **Yes** | **5.706 (1.309-24.863)**  **p=0.020** | 4.231 (0.812-22.055)  p=0.087 |
| **Lesion size (cm)** | 1.005 (0.991-1.019)  p=0.504 |  |
| **Pathological diagnosis**  **Benign lesions (reference)**  **Pheochromocytomas**  **Adrenocortical neoplasms of uncertain malignant potential**  **Adrenocortical carcinomas**  **Adrenal metastases**  **Other malignancies** | **p=0.125**  **0.741 (0.202-2.721)**  **p=0.651**  ***Not estimable***  **6.369 (1.365-39.715)**  **p=0.018**  ***Not estimable***  **10.615 (0.627-179.798)**  **p=0.102** | p=0.644  0.631 (0.149-2.670)  p=0.532  *Not estimable*  1.725 (0.162-18.327)  p=0.651  *Not estimable*  10.414 (0.512-211.817)  p=0.127 |

ICU, Intensive Care Unit.

The values reported in the table are odds ratios, with 95% confidence intervals and corresponding p-values.

Variables not retained in the final multivariable model are left blank. In the univariable analysis, bold entries identify candidate variables meeting the predefined p<0.20 criterion for inclusion in the multivariable model; in the multivariable analysis, bold entries indicate statistically significant associations at p<0.05.

* Sex as assigned at birth.

**Table S9.** Univariable and multivariable logistic regression analyses of factors associated with 30-day postoperative complications in patients undergoing unilateral adrenalectomy

|  | **Univariable analysis** | **Multivariable analysis** |
| --- | --- | --- |
| **Age (years)** | **1.018 (0.998-1.039)**  **p=0.081** | 1.007 (0.984-1.030)  p=0.560 |
| **Sex***  **Male (reference)**  **Female** | 1.270 (0.724-2.226)  p=0.405 |  |
| **BMI (kg/m^2^)** | 1.020 (0.975-1.068)  p=0.386 |  |
| **ASA score**  **1 (reference)**  **2**  **3** | **p=0.007**  **1.377 (0.469-4.040)**  **p=0.560**  **1.723 (0.874-3.396)**  **p=0.116** | p=0.059  0.818 (0.269-2.493)  p=0.724  1.924 (0.956-3.871)  p=0.067 |
| **Pattern of hormonal hypersecretion**  **Non-functioning (reference)**  **Primary hyperaldosteronism**  **Catecholamine hypersecretion**  **Hypercortisolism** | **p=0.130**  **0.191 (0.025-1.434)**  **p=0.108**  **1.019 (0.489-2.123)**  **p=0.959**  **1.545 (0.862-2.768)**  **p=0.144** | p=0.058  0.361 (0.047-2.778)  p=0.328  0.974 (0.424-2.334)  p=0.950  **2.047 (1.079-3.884)**  **p=0.028** |
| **Previous abdominal surgery**  **No (reference)**  **Yes** | **1.744 (1.035-2.937)**  **p=0.037** | 1.710 (0.974-3.001)  p=0.062 |
| **Operative time (minutes)** | **1.012 (1.007-1.017)**  **p<0.001** | **1.009 (1.002-1.015)**  **p=0.006** |
| **Side**  **Right (reference)**  **Left** | **1.648 (0.968-2.805)**  **p=0.066** | 1.334 (0.755-2.358)  p=0.321 |
| **Surgical procedure**  **Laparoscopic LTA (reference)**  **Laparoscopic PRA**  **Robotic LTA**  **Robotic PRA** | p=0.896  0.915 (0.453-1.848)  p=0.805  1.118 (0.652-2.165)  p=0.574  1.244 (0.276-5.617)  p=0.776 |  |
| **Conversion**  **No (reference)**  **Yes** | **7.302 (3.080-17.310)**  **p<0.001** | **3.145 (1.096-9.030)**  **p=0.033** |
| **Partial adrenalectomy**  **No (reference)**  **Yes** | *Not estimable* |  |
| **Lymph node dissection**  **No (reference)**  **Yes** | **2.526 (1.124-5.677)**  **p=0.025** | 0.838 (0.282-2.488)  p=0.750 |
| **Multiorgan resection**  **No (reference)**  **Yes** | **9.333 (3.603-24.179)**  **p<0.001** | **3.738 (1.181-11.838)**  **p=0.025** |
| **Intraoperative blood transfusion**  **No (reference)**  **Yes** | **35.746 (3.661-349.024)**  **p=0.002** | 3.939 (0.148-105.106)  p=0.413 |
| **Intraoperative complications**  **No (reference)**  **Yes** | **2.787 (0.908-8.556)**  **p=0.073** | 0.391 (0.084-1.812)  p=0.230 |
| **ICU stay**  **No (reference)**  **Yes** | **5.264 (2.308-12.004)**  **p<0.001** | 2.423 (0.922-6.370)  p=0.073 |
| **Lesion size (cm)** | 1.002 (0.993-1.011)  p=0.654 |  |
| **Pathological diagnosis**  **Benign lesions (reference)**  **Pheochromocytomas**  **Adrenocortical neoplasms of uncertain malignant potential**  **Adrenocortical carcinomas**  **Adrenal metastases**  **Other malignancies** | p=0.229  1.309 (0.687-2.493)  p=0.413  2.085 (0.453-9.599)  p=0.345  3.389 (1.202-9.557)  p=0.021  1.643 (0.552-4.894)  p=0.373  2.711 (0.308-23.829)  p=0.368 |  |

LTA, Lateral Transabdominal Adrenalectomy; PRA, Posterior Retroperitoneoscopic Adrenalectomy; ICU, Intensive Care Unit.

The values reported in the table are odds ratios, with 95% confidence intervals and corresponding p-values.

Variables not retained in the final multivariable model are left blank. In the univariable analysis, bold entries identify candidate variables meeting the predefined p<0.20 criterion for inclusion in the multivariable model; in the multivariable analysis, bold entries indicate statistically significant associations at p<0.05.

* Sex as assigned at birth.

**Table S10.** Sensitivity analyses of factors associated with 30-day postoperative complications after adjustment for temporal heterogeneity

| **Variable** | **Model adjusted for surgical era OR (95% CI), p-value** | **Model adjusted for chronological case order OR (95% CI), p-value** |
| --- | --- | --- |
| **ASA score  1 (reference)  2  3** | p=0.054  0.638 (0.246-1.658), p=0.297 **1.893 (1.152-3.316), p=0.035** | **p=0.049**  0.678 (0.275-1.763), p=0.104 **1.967 (1.068-3.442), p=0.031** |
| **Pattern of hormonal hypersecretion  Non-functioning (reference)  Primary hyperaldosteronism  Catecholamine hypersecretion  Hypercortisolism** | **p=0.006**  0.309 (0.040-2.369), p=0.258 0.845 (0.371-1.923), p=0.688 **2.313 (1.263-4.235), p=0.007** | **p=0.005**  0.305 (0.040-2.341), p=0.253 0.854 (0.376-1.941), p=0.707 **2.358 (1.288-4.318), p=0.005** |
| **Operative time (minutes)** | **1.008 (1.002-1.013), p=0.007** | **1.007 (1.002-1.013), p=0.009** |
| **Conversion  No (reference) Yes** | **2.990 (1.064-8.402), p=0.038** | **3.051 (1.087-8.560), p=0.034** |
| **ICU stay  No (reference)  Yes** | **2.335 (1.004-5.427), p=0.049** | 2.168 (0.933-5.038), p=0.072 |
| **Multiorgan resection  No (reference)  Yes** | Reference **4.662 (1.441-15.086), p=0.010** | Reference **4.886 (1.495-15.963), p=0.009** |
| **Temporal covariate  Late era (2019 onwards vs up to 2018)  Chronological case order** | 0.626 (0.359-1.092), p=0.099 - | - 0.999 (0.998-1.000), p=0.084 |

Values are presented as adjusted odds ratios with 95% confidence intervals and corresponding p-values. The surgical-era model compared procedures performed from 2019 onwards with those performed up to and including 2018, according to the inflection identified by cumulative sum analysis. Chronological case order was entered as a continuous covariate ranging from 1 to 798. ICU, Intensive Care Unit. Bold entries indicate statistically significant associations or overall tests at p<0.05.

**Detailed description of intraoperative complications stratified by surgical procedure**

Overall, 21 intraoperative complications were recorded among 798 minimally invasive adrenalectomies (2.6%). According to surgical procedure, 10 events occurred during laparoscopic lateral transabdominal adrenalectomy, five during laparoscopic posterior retroperitoneoscopic adrenalectomy, five during robotic lateral transabdominal adrenalectomy, and one during robotic posterior retroperitoneoscopic adrenalectomy. During laparoscopic lateral transabdominal adrenalectomy, reported events included haemodynamic instability (n=1), inferior vena cava bleeding (n=4), duodenal injury (n=1), splenic laceration (n=1), adrenal vein bleeding (n=1), right renal vein bleeding (n=1), and left renal artery bleeding (n=1). During laparoscopic posterior retroperitoneoscopic adrenalectomy, complications comprised haemodynamic instability (n=1), inferior vena cava bleeding (n=1), renal parenchymal bleeding (n=2), and left colonic injury (n=1). In the robotic lateral transabdominal adrenalectomy group, reported events were adrenal vein bleeding (n=3), hepatic parenchymal bleeding (n=1), and right renal vein bleeding (n=1). Robotic posterior retroperitoneoscopic adrenalectomy was associated with one case of inferior vena cava bleeding.

**Detailed description of 30-day postoperative complications according to surgical procedure**

Overall, 70 patients developed 30-day postoperative complications (8.8%). Twenty-nine complications occurred after laparoscopic lateral transabdominal adrenalectomy, 19 after laparoscopic posterior retroperitoneoscopic adrenalectomy, 20 after robotic lateral transabdominal adrenalectomy, and two after robotic posterior retroperitoneoscopic adrenalectomy.

Following laparoscopic lateral transabdominal adrenalectomy, reported complications included abdominal pain (1 grade I), postoperative elevation of liver enzymes (1 grade I), pleural effusion (1 grade I), pancreatitis (1 grade I), polyuria (1 grade II), fever (2 patients, both grade II), pneumonia (4 patients, all grade II), atrial fibrillation (3 patients, all grade II), urinary tract infection (1 grade II), bleeding (2 patients, both grade II), intraparenchymal renal bleeding (1 grade II), renal ischaemia (1 grade II), lymphatic fistula (2 patients, both grade II), sepsis due to Staphylococcus aureus (1 grade II), non-ST-elevation myocardial infarction (1 grade II), acute respiratory failure (2 patients, both grade IV), trocar-site bleeding (1 grade IIIb), perforated gastric ulcer (1 grade IIIb and 1 grade IV), and splenic rupture (1 grade IV).

After laparoscopic posterior retroperitoneoscopic adrenalectomy, complications comprised haematuria (1 grade I), neutropenia (1 grade I), superficial surgical site infection (1 grade II), fever (4 patients, all grade II), pneumonia (3 patients, all grade II), lymphatic fistula (1 grade II), urinary tract infection (1 grade II), lymphatic fistula with superficial surgical site infection (1 grade II), bleeding (1 grade II), subcapsular hepatic haematoma with arterioportal fistula (1 grade IIIa), intraparenchymal renal bleeding with lymphatic fistula (1 grade IIIa), bleeding (2 patients, both grade IV), and haemoperitoneum (1 grade V).

In the robotic lateral transabdominal adrenalectomy group, postoperative elevation of liver enzymes (1 grade I), adrenal bed haematoma (1 grade I), pancreatitis (1 grade I), pleural effusion (3 patients: 1 grade I and 2 grade II), fever (4 patients: 2 grade I and 2 grade II), pneumonia (3 patients, all grade II), trocar-site bleeding (1 grade IIIb), superficial surgical site infection (2 patients, both grade II), pancreatitis (1 grade II), bleeding (1 grade II and 1 grade IV), and wall haematoma (1 grade I) were observed.

Finally, robotic posterior retroperitoneoscopic adrenalectomy was associated with pleural effusion (1 grade I) and acute myocardial infarction (1 grade V).

**Detailed description of postoperative mortality within 6 months after surgery**

Four deaths occurred within 6 months after surgery. Two patients died within the first 30 postoperative days: one patient

who had undergone bilateral laparoscopic posterior retroperitoneoscopic adrenalectomy for ACTH-dependent Cushing syndrome secondary to ectopic hormone secretion died on postoperative day 1 from metabolic complications following haemorrhagic shock, whereas one patient who had undergone robotic posterior retroperitoneoscopic adrenalectomy for ACTH-independent Cushing syndrome died from myocardial infarction 3 weeks after surgery. Two additional deaths occurred thereafter within the 6-month postoperative period: one patient who had undergone laparoscopic lateral transabdominal adrenalectomy died 2 months after surgery due to severe respiratory failure, whereas one patient who had undergone bilateral laparoscopic posterior retroperitoneoscopic adrenalectomy for ACTH-dependent Cushing syndrome caused by ectopic hormone secretion from a lung neuroendocrine tumour died 5 months after surgery from metabolic complications related to systemic chemotherapy.

**Figure legends**

**Figure S1.** Receiver operating characteristic curve of the final multivariable logistic regression model for 30-day postoperative complications.

**Figure S2.** Observed 30-day postoperative complication rates across tertiles of model-derived predicted risk.

Patients were stratified according to tertiles of predicted probability derived from the final multivariable logistic regression model. Observed complication rates increased progressively across predicted-risk tertiles: 7/266 (2.6%) in the low-risk tertile, 14/266 (5.3%) in the intermediate-risk tertile, and 49/266 (18.4%) in the high-risk tertile (p for trend <0.001).

**Figure S3.** Recursive cumulative sum analysis of 30-day postoperative complications across the overall minimally invasive adrenalectomy series.

A potential temporal inflection point was identified around the 221st case (early 2018), followed by a more stable complication pattern over time.

**Figure S4.** Temporal trend of 30-day postoperative complications across consecutive minimally invasive adrenalectomy cases.

No significant linear decline in complication rate was observed throughout the study period.
